# Supplementary material for: Seven New Phenylhexanoids with Antioxidant Activity from Saxifraga umbellulata var. pectinata
Source: Molecules. 2023 May 6;28(9):3928. doi: 10.3390/molecules28093928 (PMC10180102; doi:10.3390/molecules28093928)
Supplement: Supplementary file 1 [file molecules-28-03928-s001.zip › molecules-2315601-supplementary.pdf]

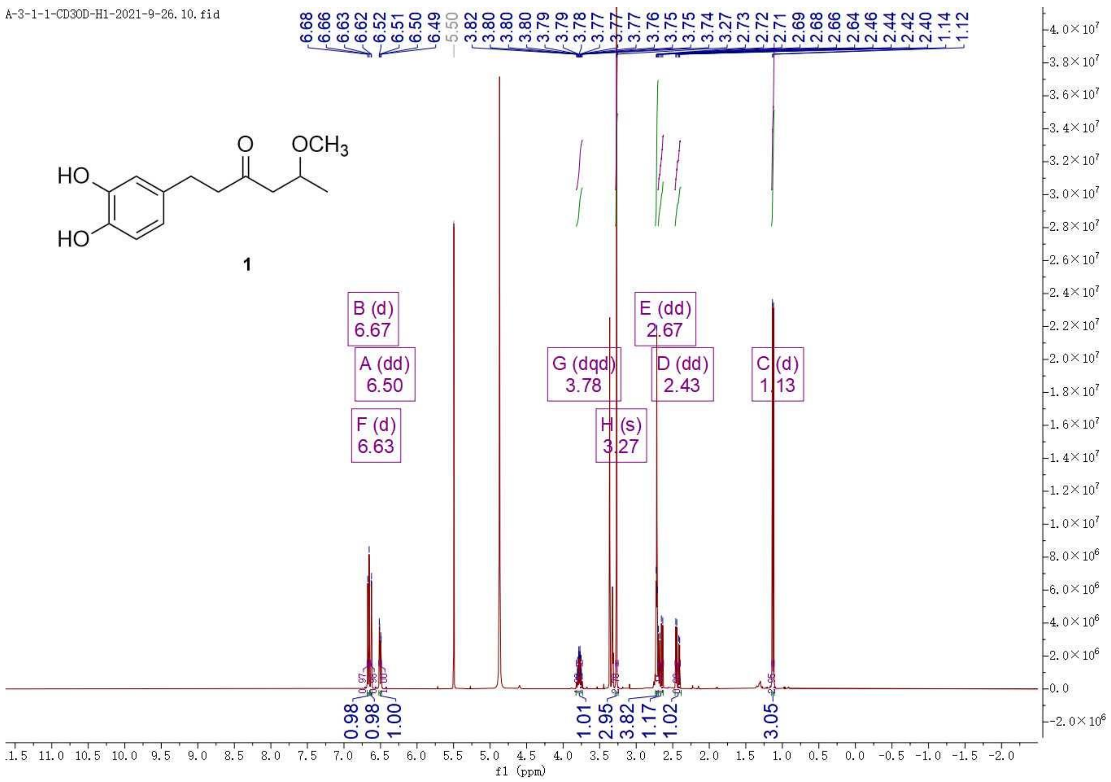

Figure S1-1. ^{1}H-NMR spectrum (400 MHz) of **1** in CD_3OD

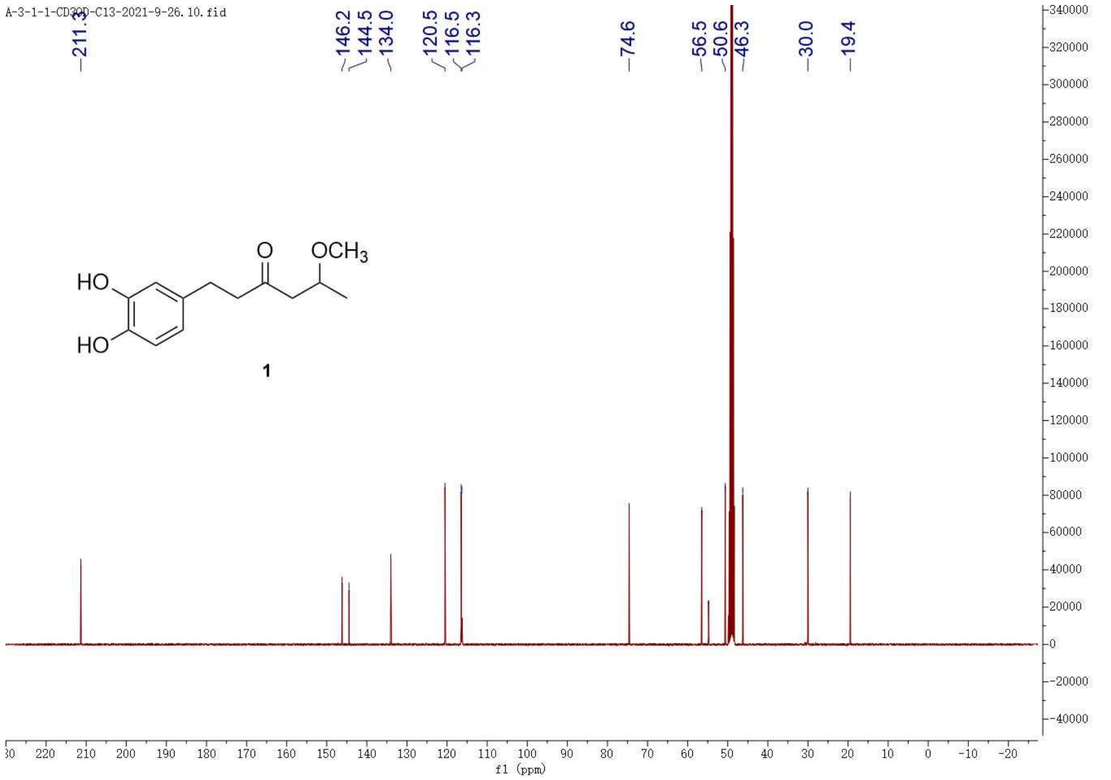

Figure S1-2. ^{13}C-NMR spectrum (100 MHz) of **1** in CD_3OD

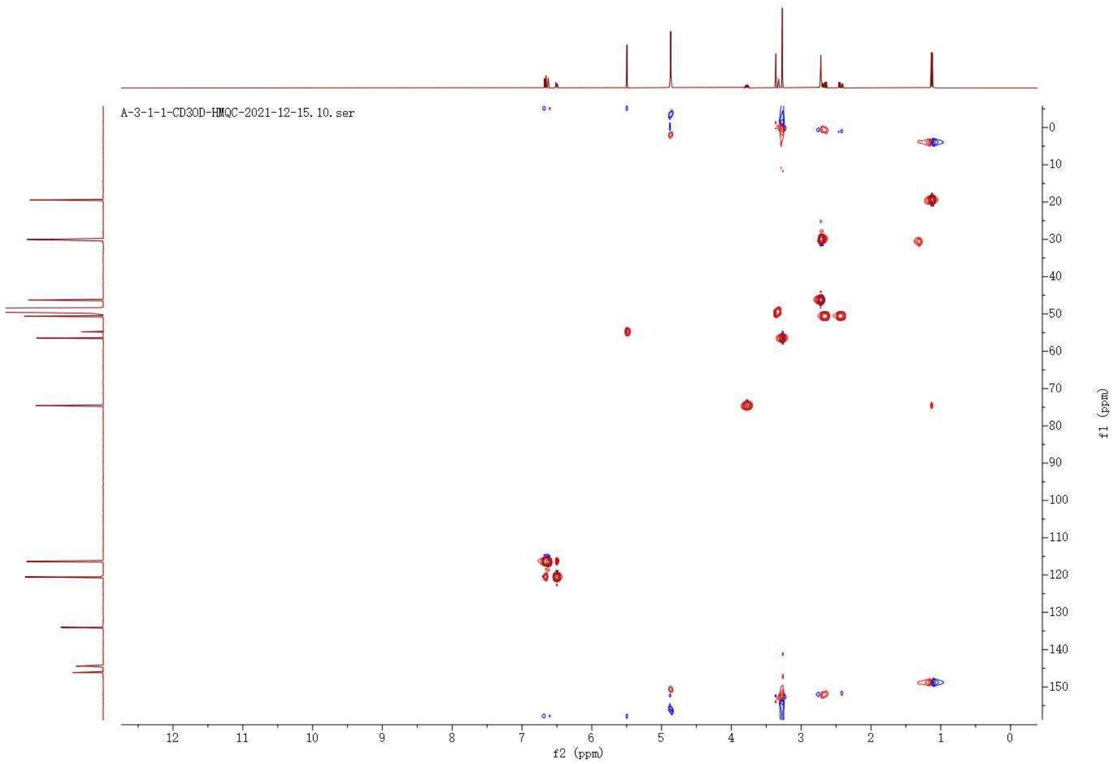

Figure S1-3. HMQC spectrum of **1** in CD_3OD

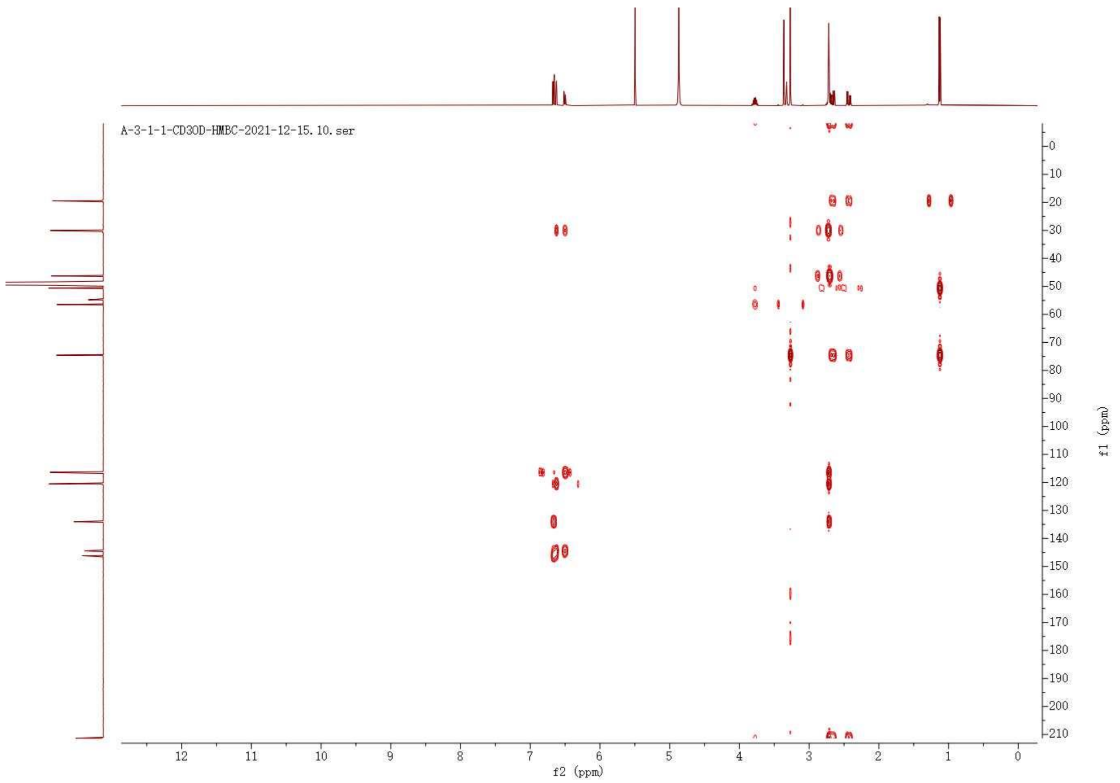

Figure S1-4. HMBC spectrum of **1** in CD_3OD

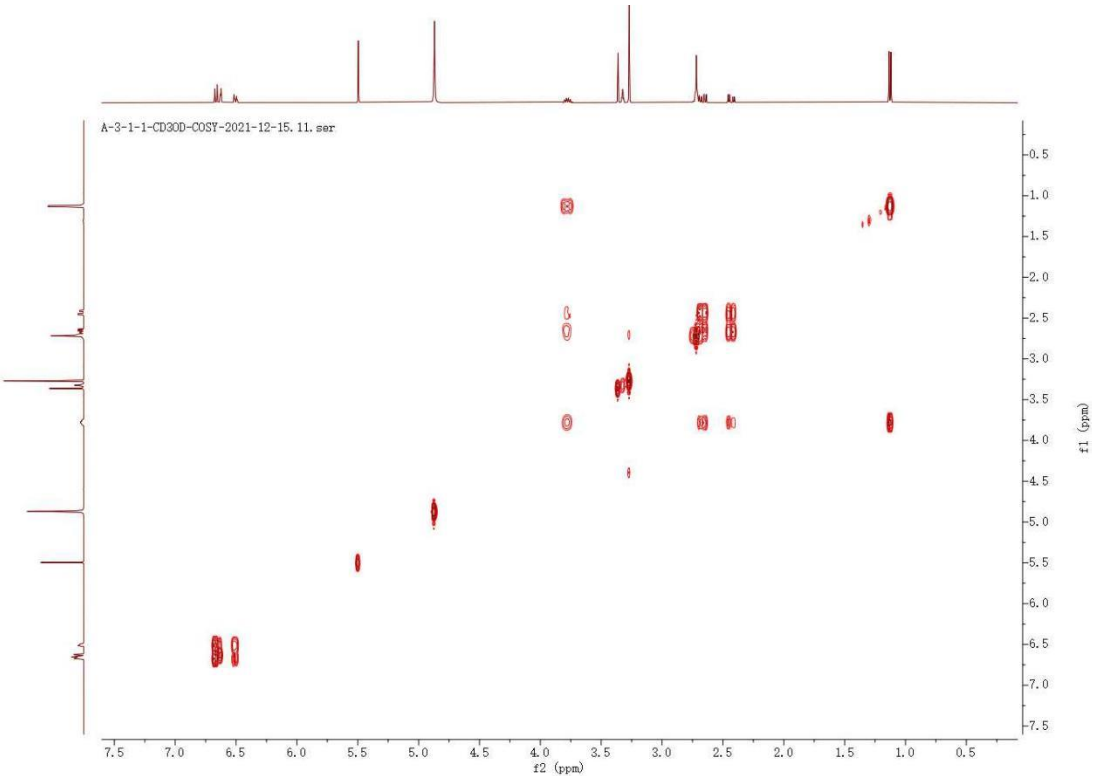

Figure S1-5. ^{1}H-^{1}H COSY spectrum of **1** in CD_3OD

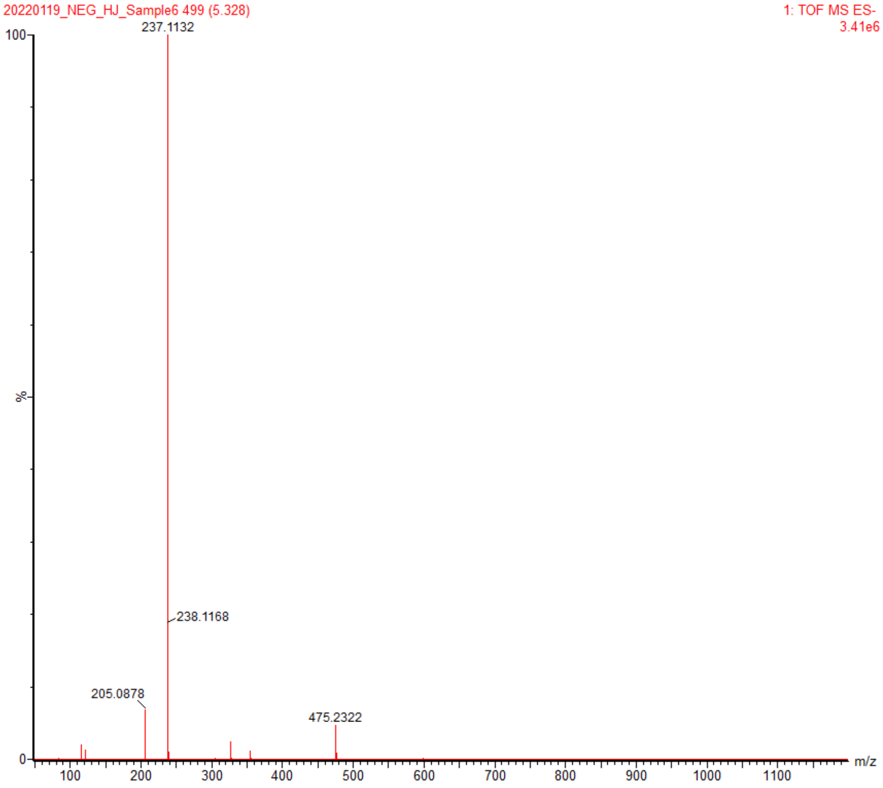

Figure S1-6. HR-ESI-MS spectrum of **1**

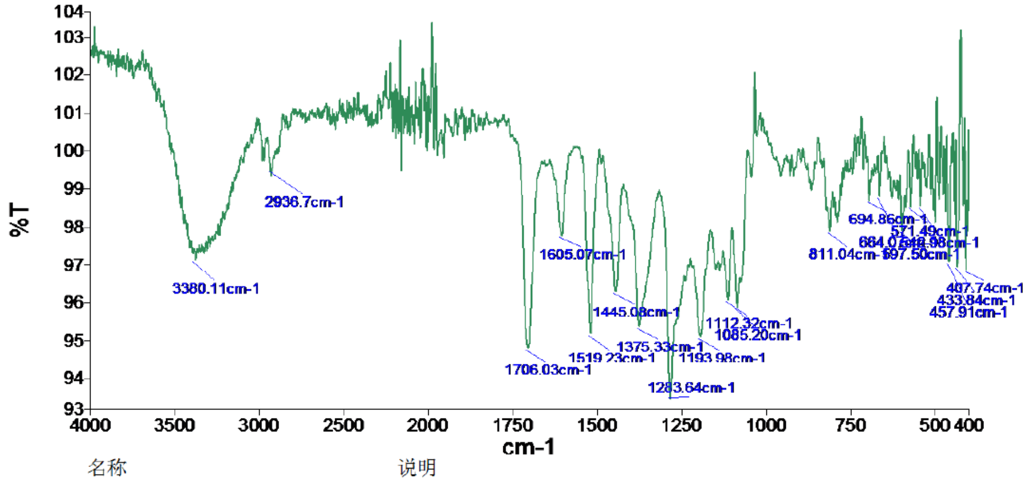

Figure S1-7. IR spectrum of compound **1** (film)

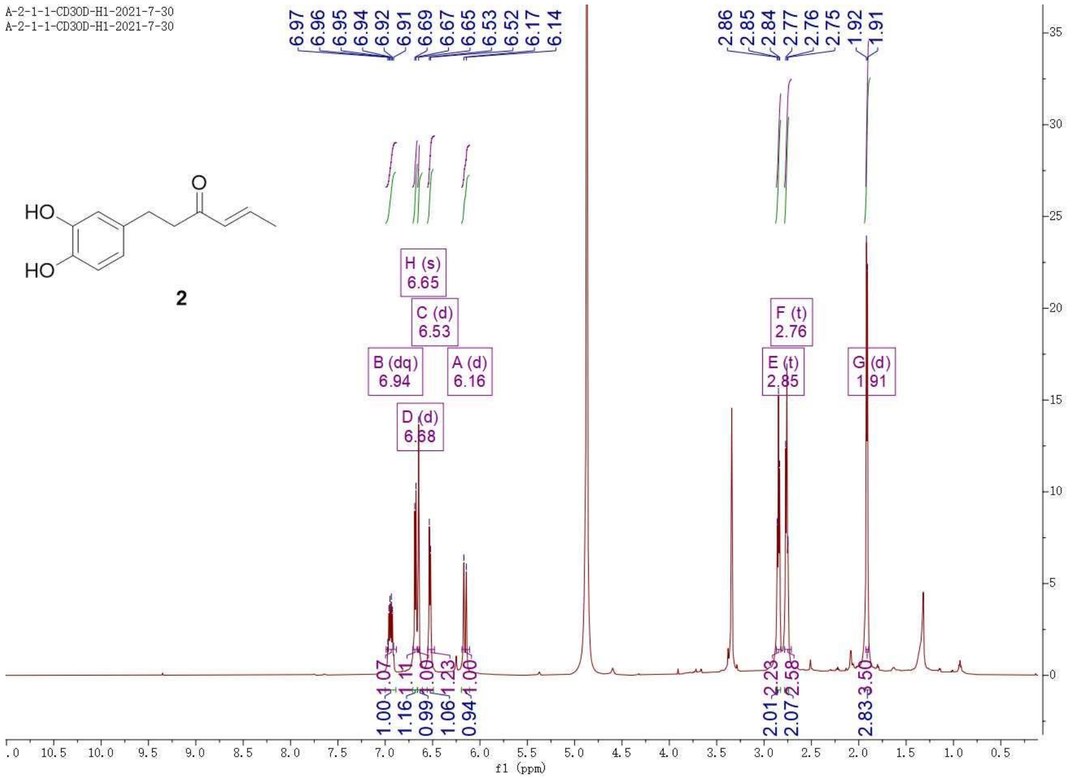

Figure S2-1. ^{1}H-NMR spectrum (400 MHz) of **2** in CD_3OD

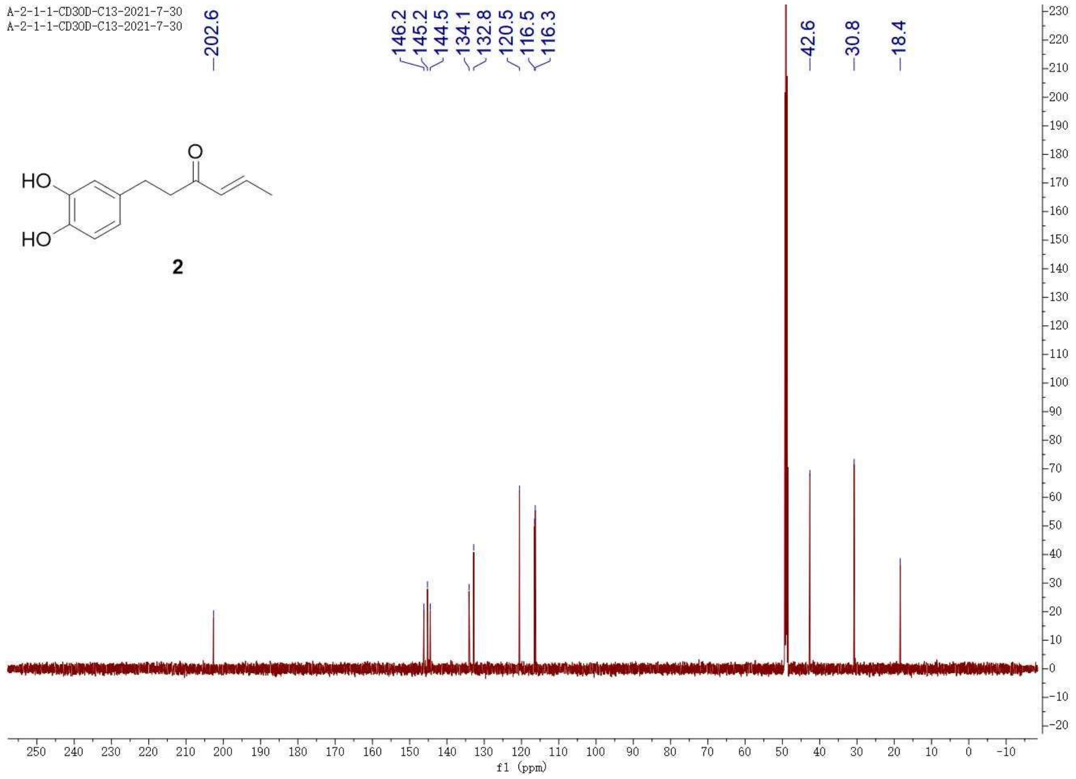

Figure S2-2. ^{13}C-NMR spectrum (100 MHz) of **2** in CD_3OD

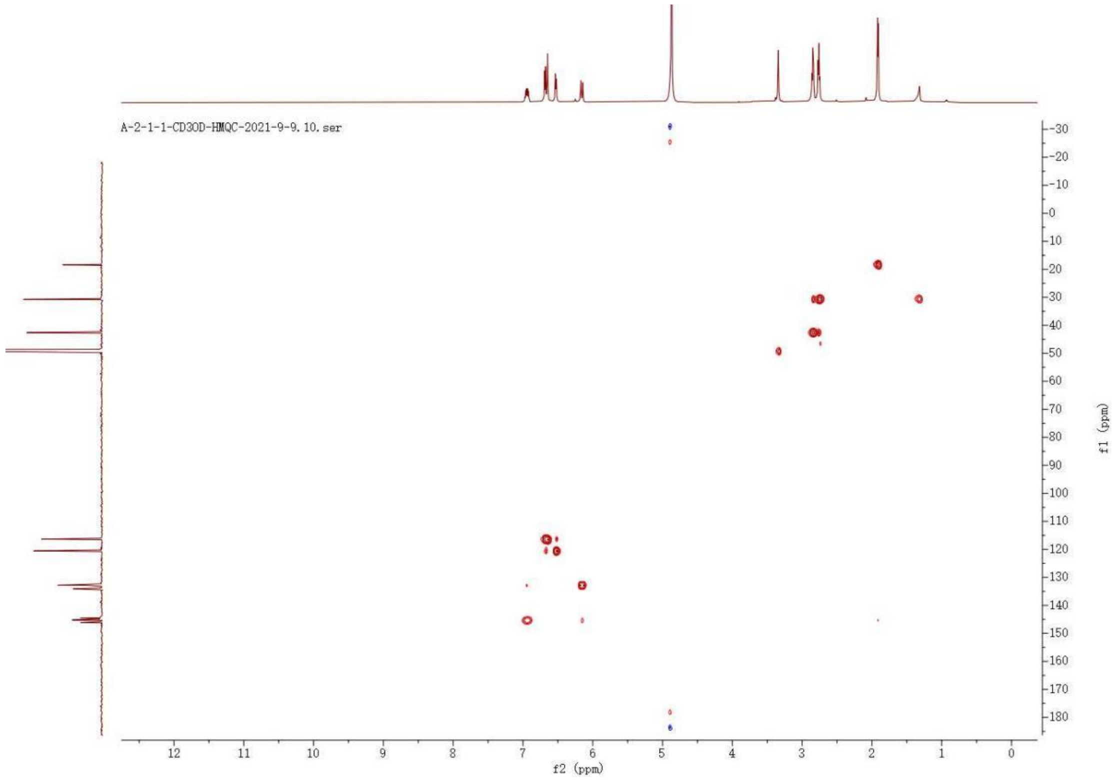

Figure S2-3. HMQC spectrum of **2** in CD_3OD

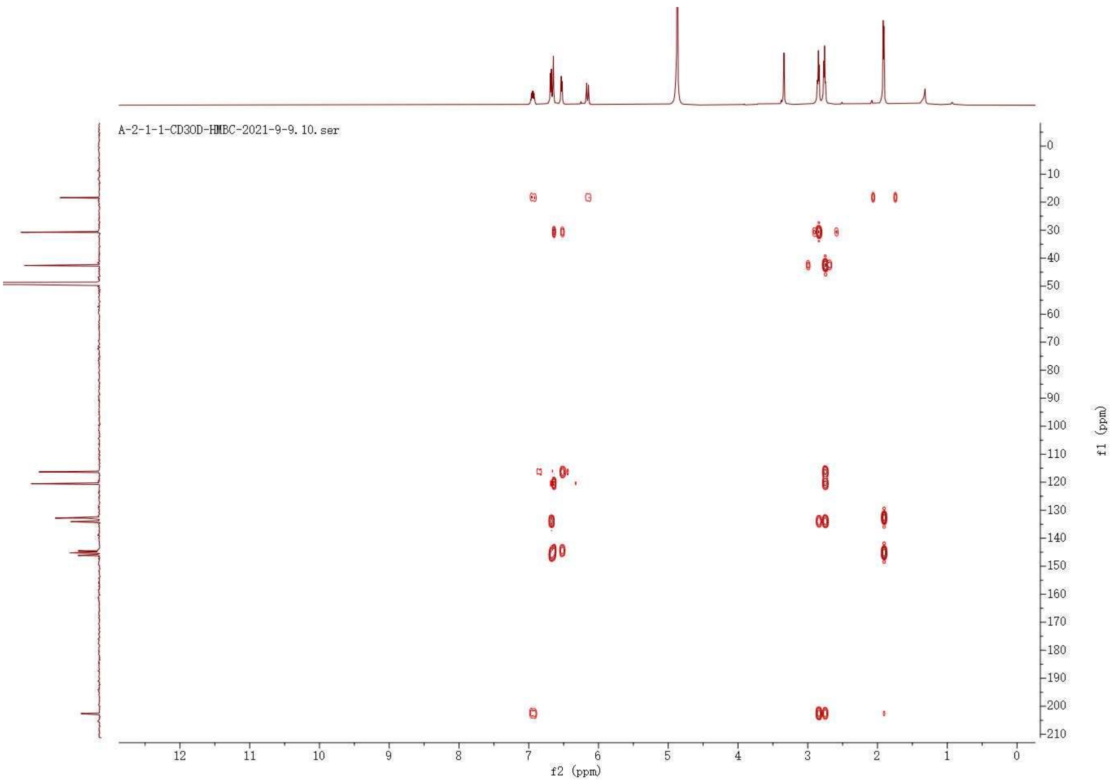

Figure S2-4. HMBC spectrum of **2** in CD_3OD

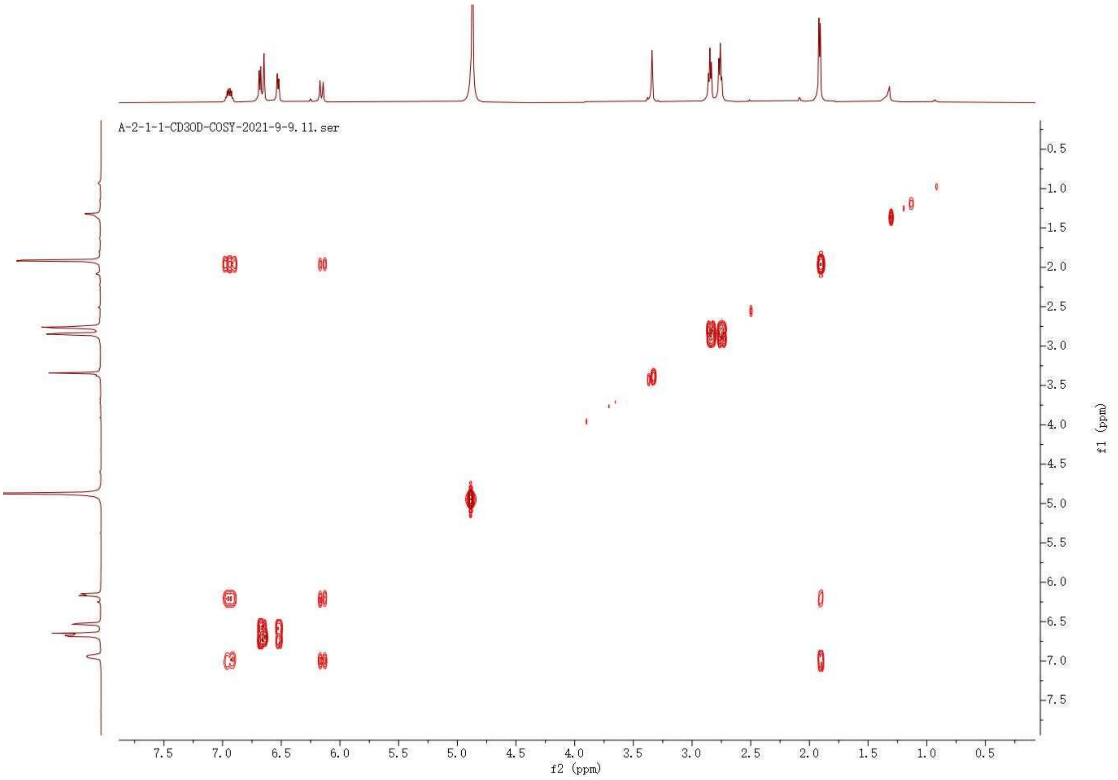

Figure S2-5. ^1H-^1H COSY spectrum of **2** in CD_3OD

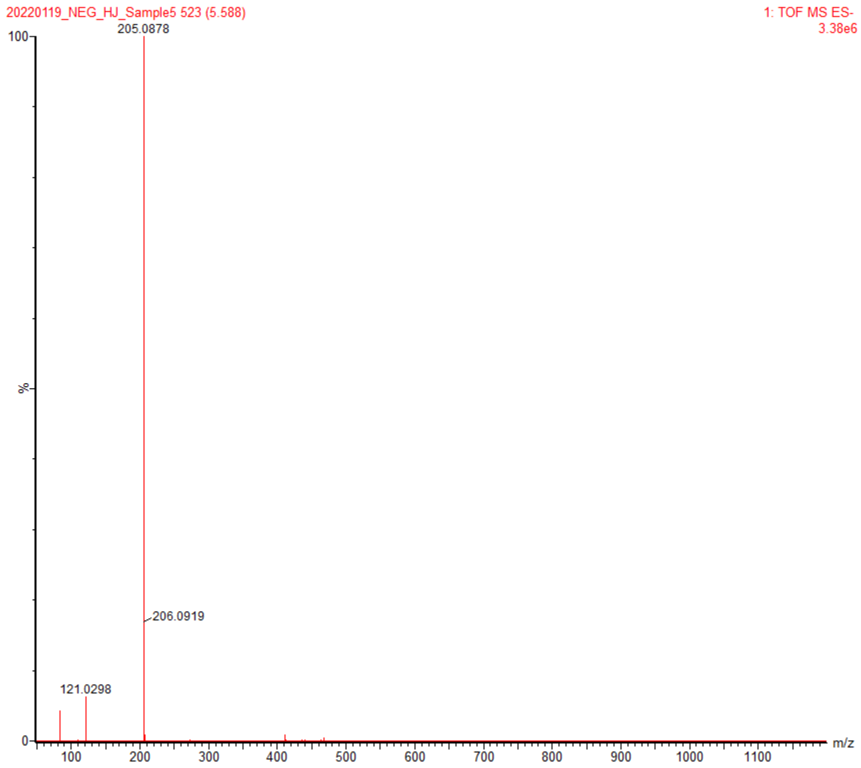

Figure S2-6. HR-ESI-MS spectrum of **2**

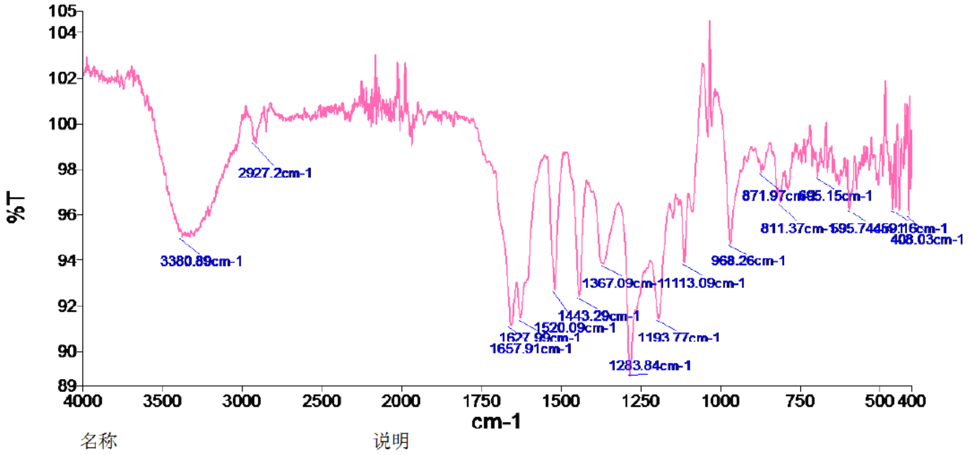

Figure S2-7. IR spectrum of compound **2** (film)

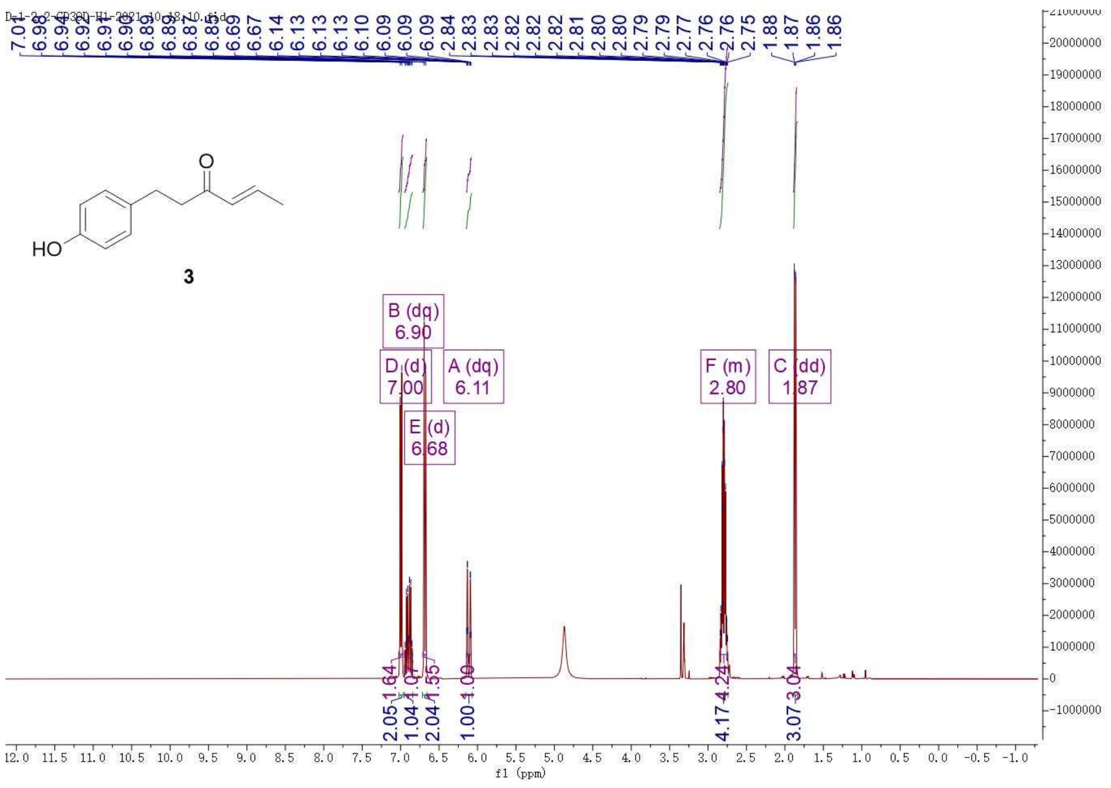

Figure S3-1. ^{1}H-NMR spectrum (400 MHz) of **3** in CD_3OD

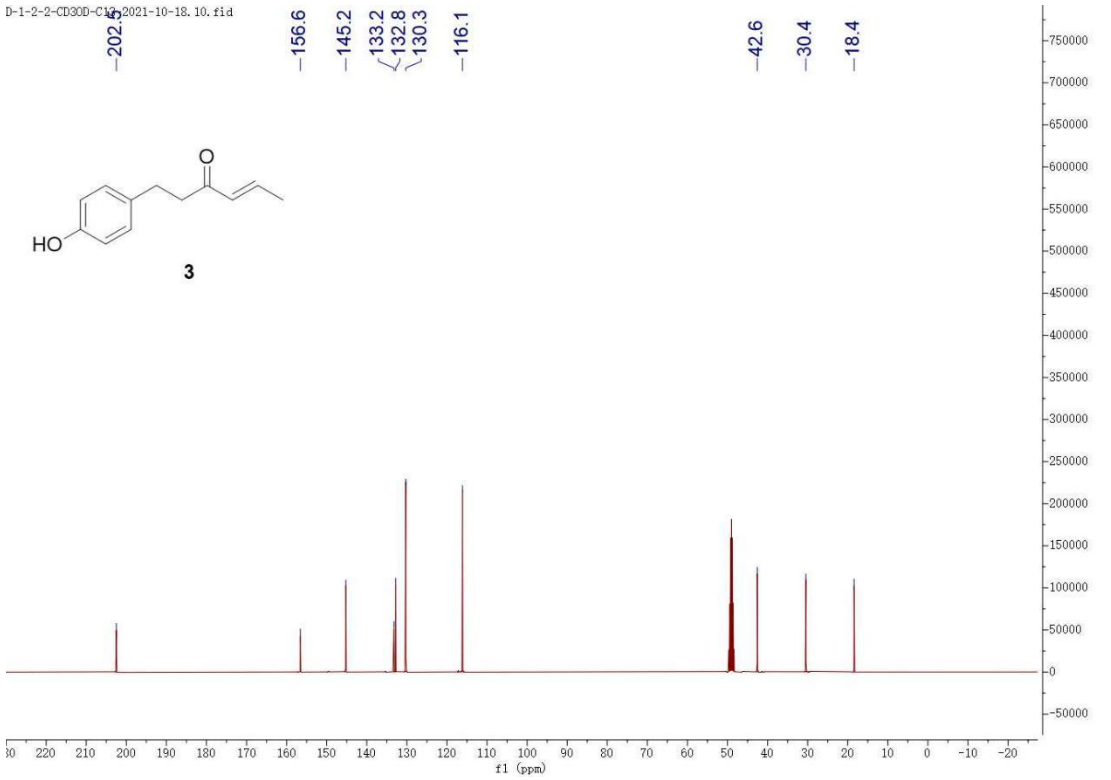

Figure S3-2. ^{13}C-NMR spectrum (100 MHz) of **3** in CD_3OD

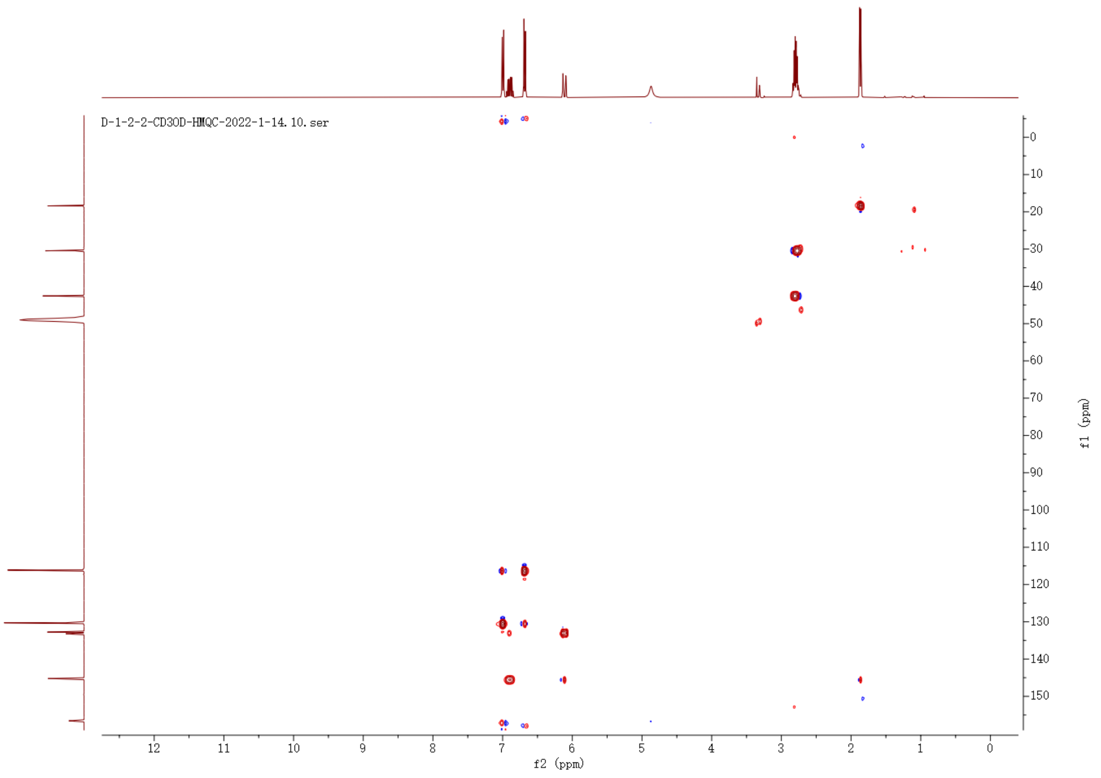

Figure S3-3. HMQC spectrum of **3** in CD_3OD

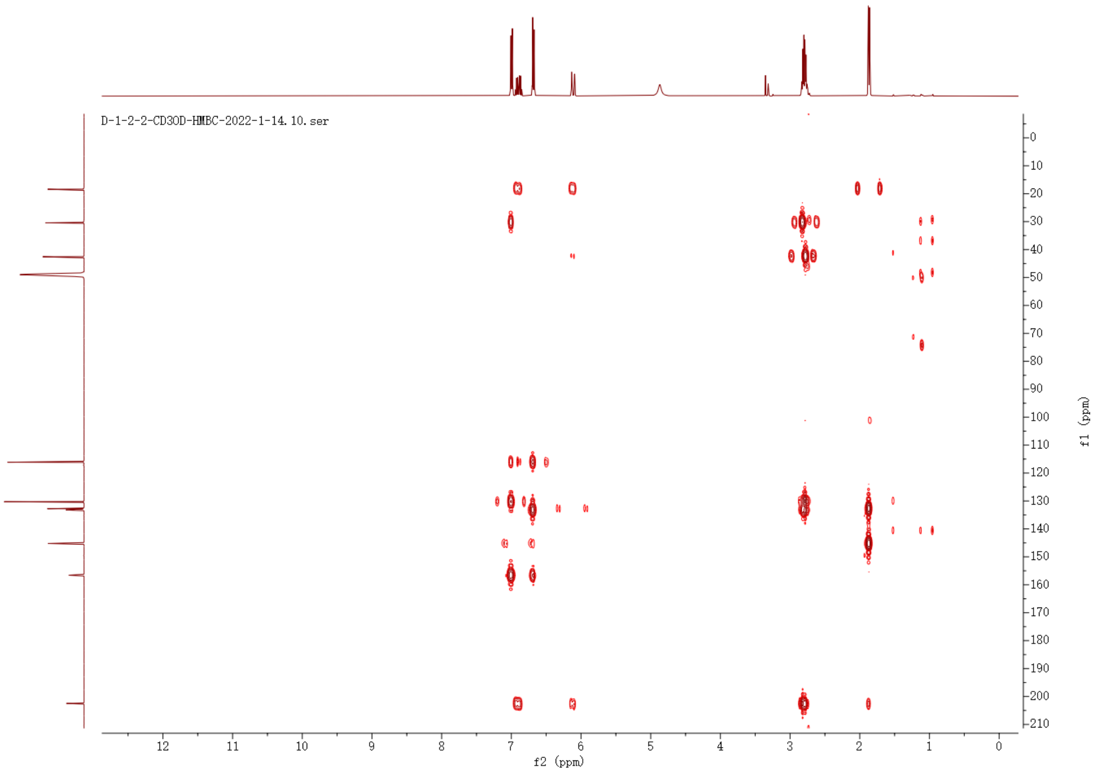

Figure S3-4. HMBC spectrum of **3** in CD_3OD

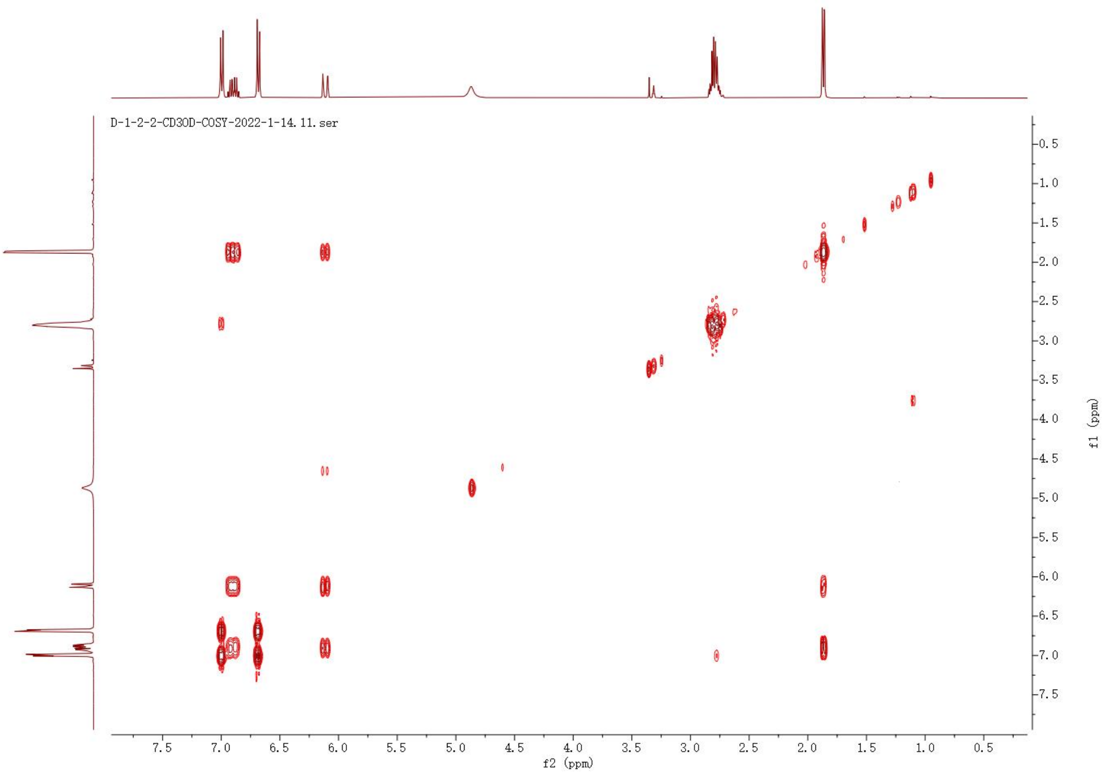

Figure S3-5. ^{1}H-^{1}H COSY spectrum of **3** in CD_3OD

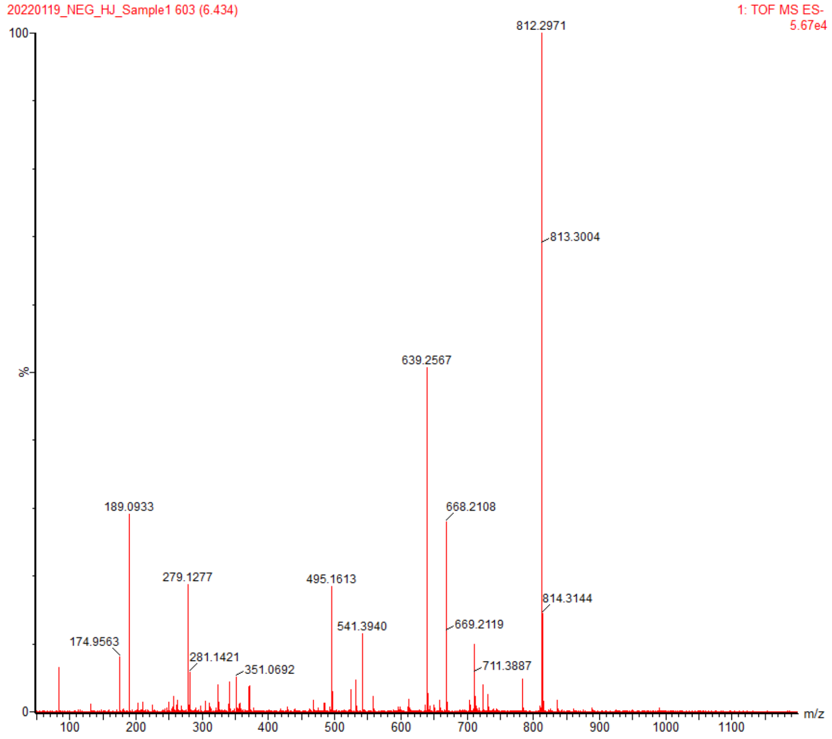

Figure S3-6. HR-ESI-MS spectrum of **3**

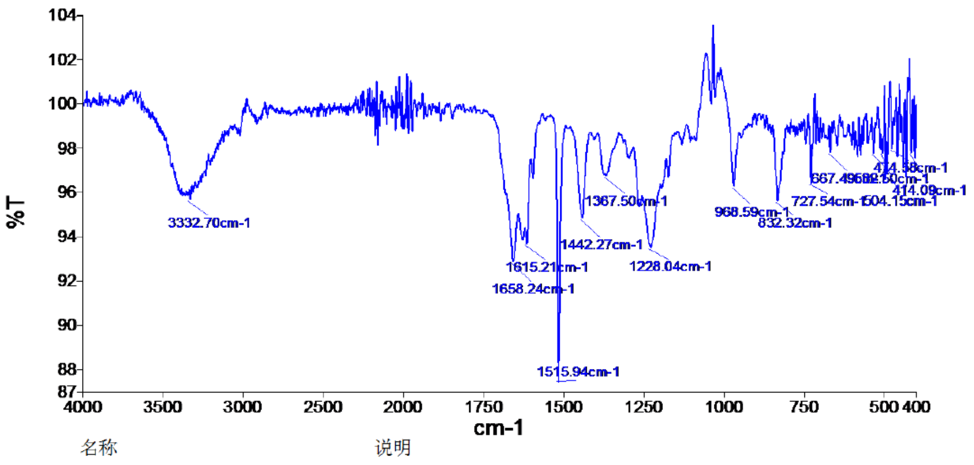

Figure S3-7. IR spectrum of compound **3** (film)

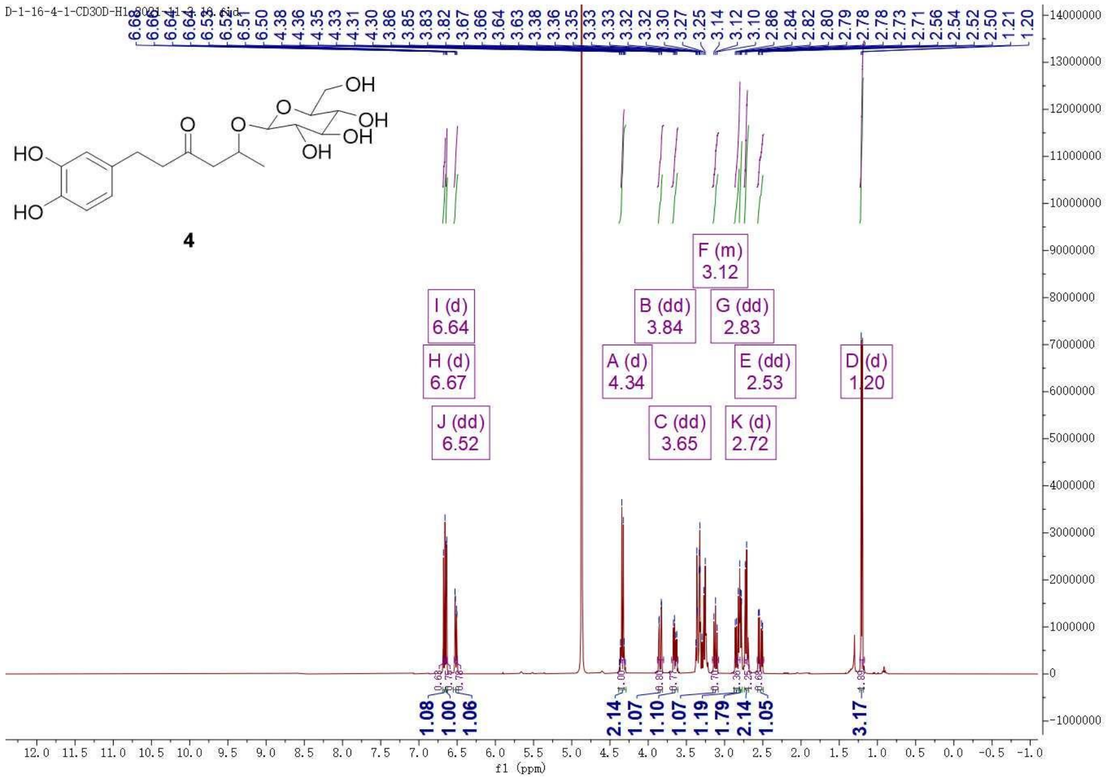

Figure S4-1. ^{1}H-NMR spectrum (400 MHz) of **4** in CD_3OD

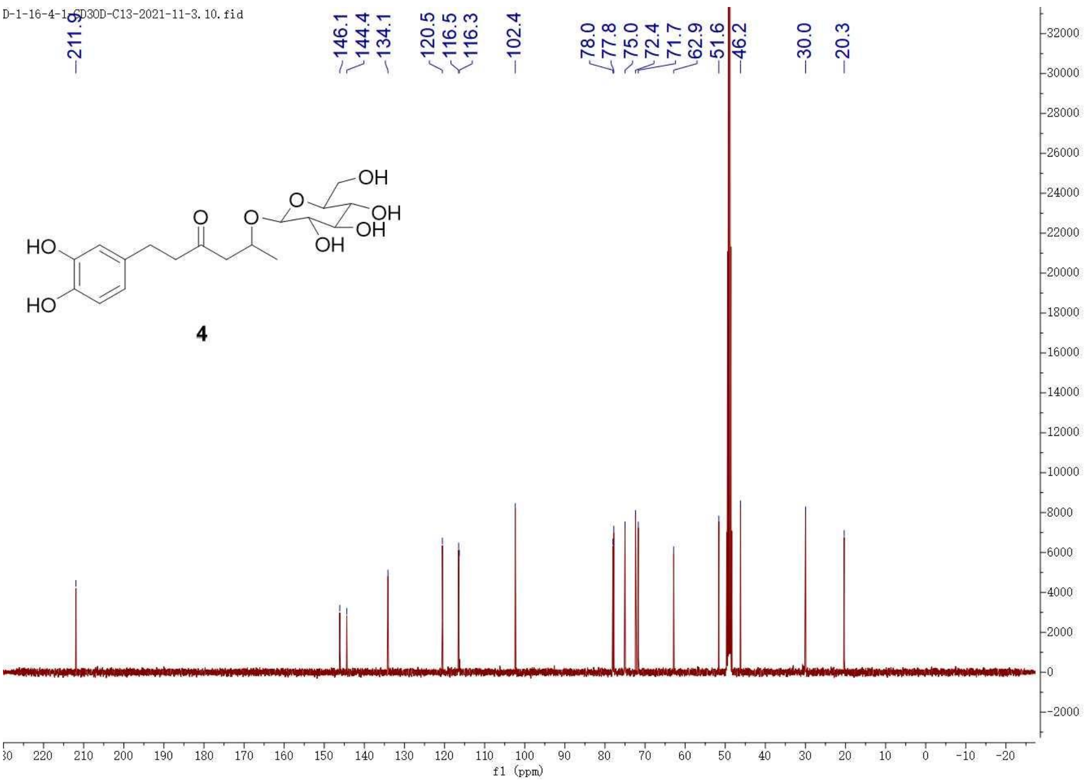

Figure S4-2. ^{13}C-NMR spectrum (100 MHz) of **4** in CD_3OD

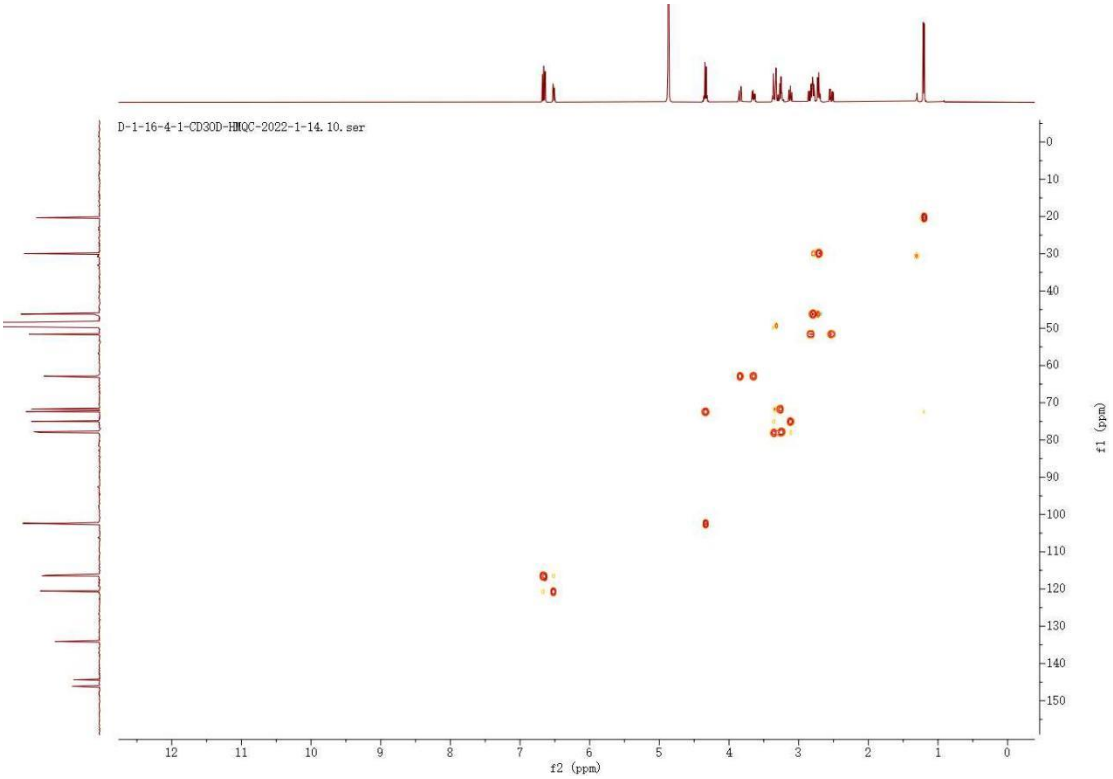

Figure S4-3. HMQC spectrum of **4** in CD_3OD

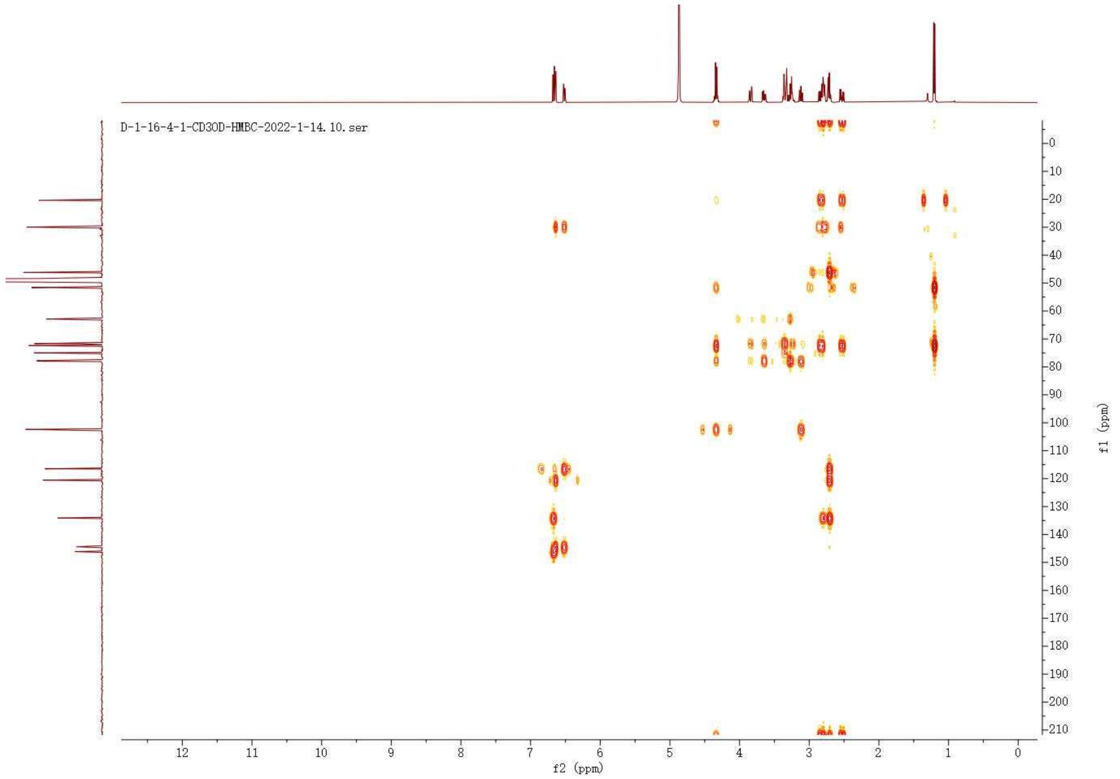

Figure S4-4. HMBC spectrum of **4** in CD_3OD

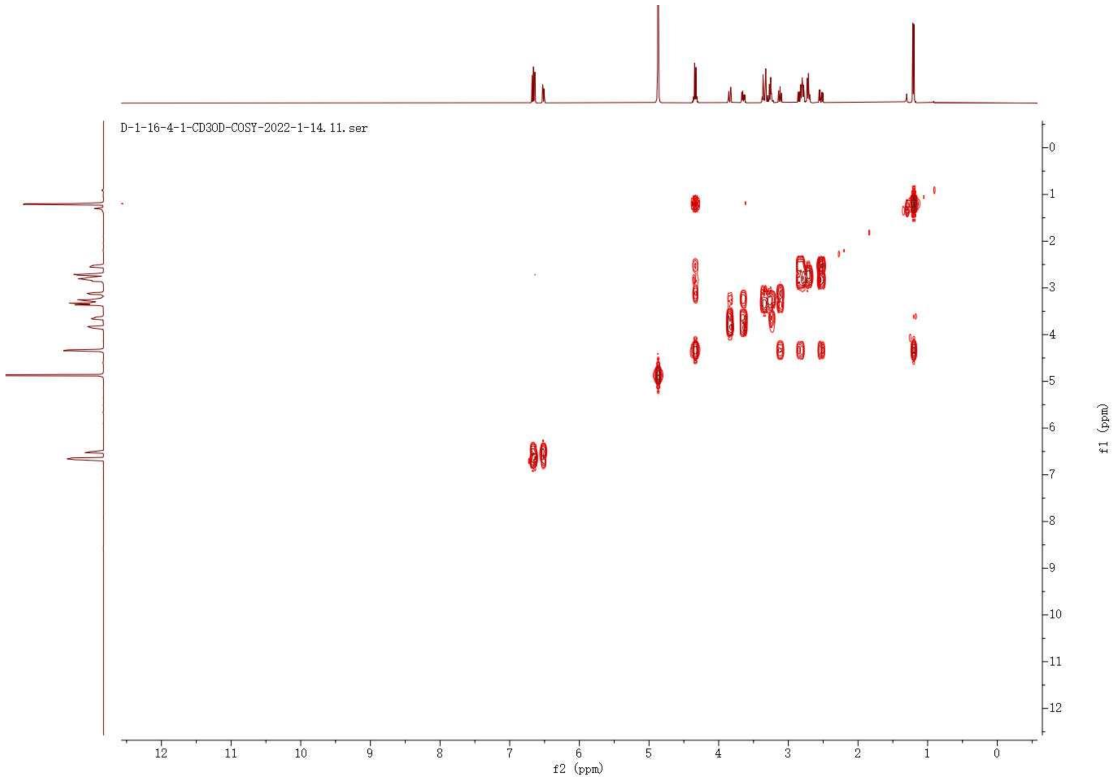

Figure S4-5. ^1H-^1H COSY spectrum of **4** in CD_3OD

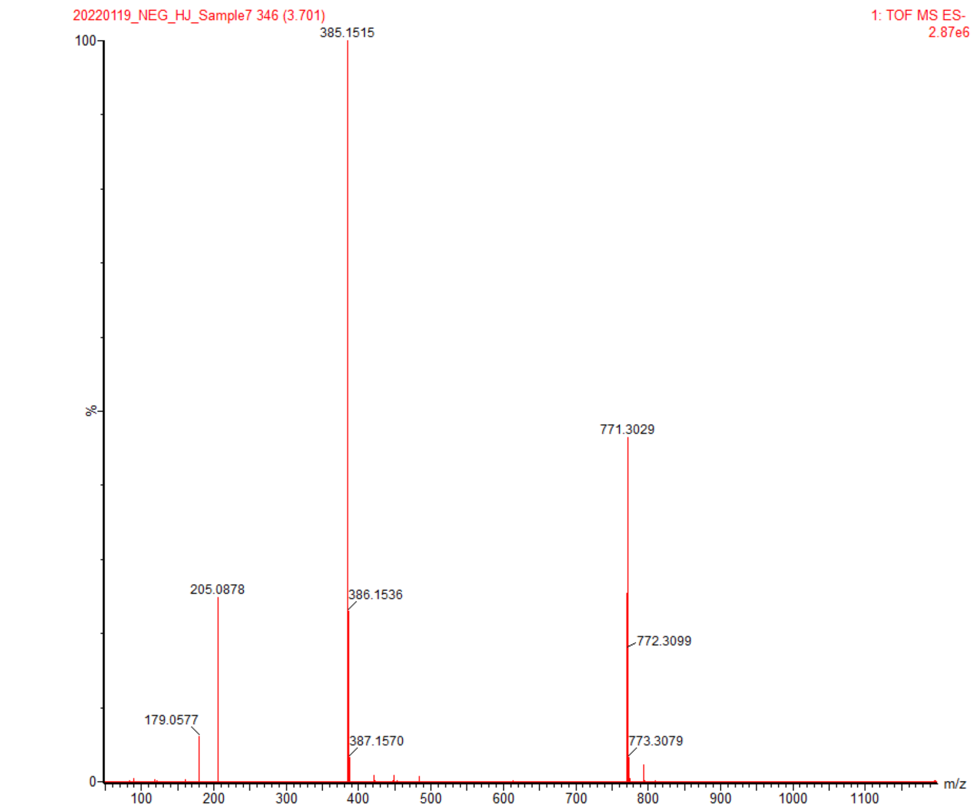

Figure S4-6. HR-ESI-MS spectrum of **4**

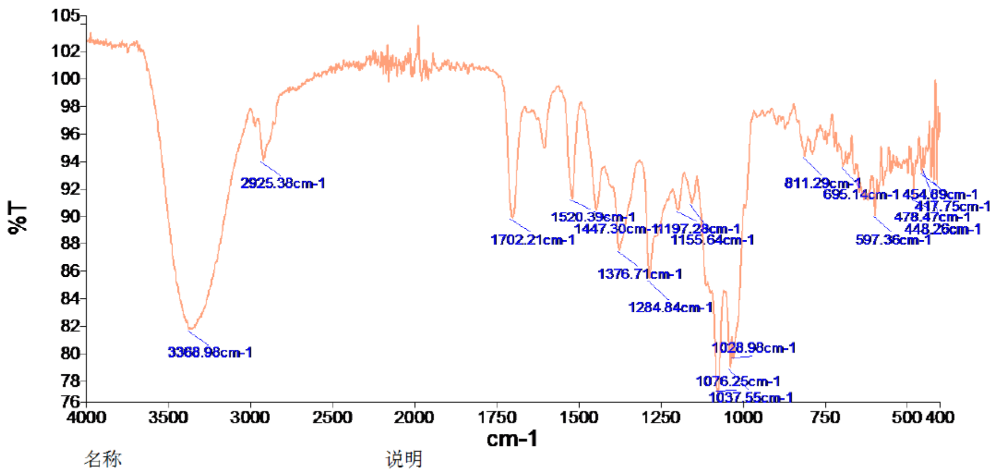

Figure S4-7. IR spectrum of compound **4** (film)

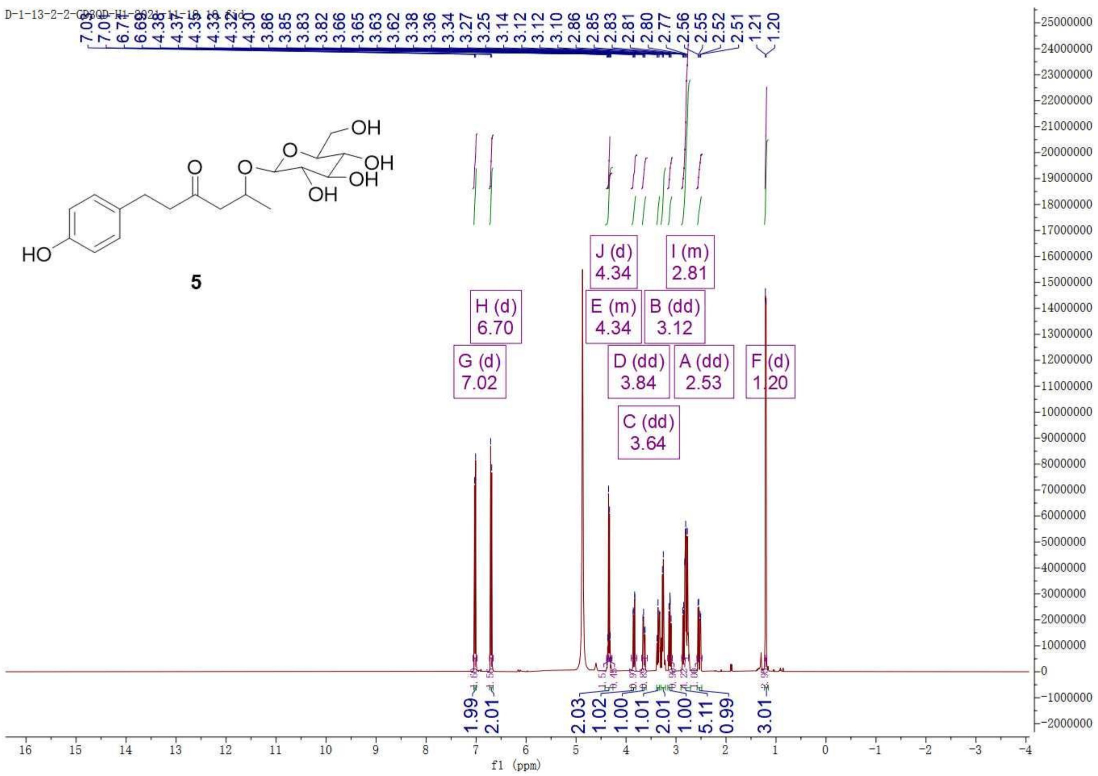

Figure S5-1. ^{1}H-NMR spectrum (400 MHz) of **5** in CD_3OD

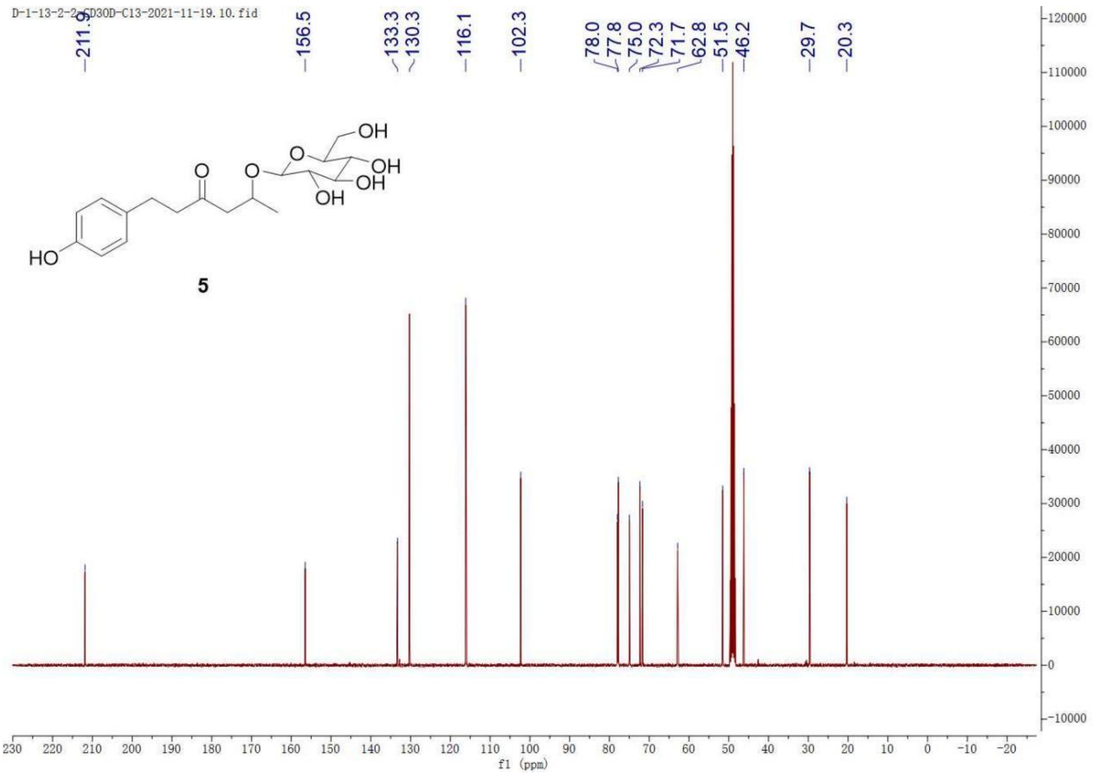

Figure S5-2. ^{13}C-NMR spectrum (100 MHz) of **5** in CD_3OD

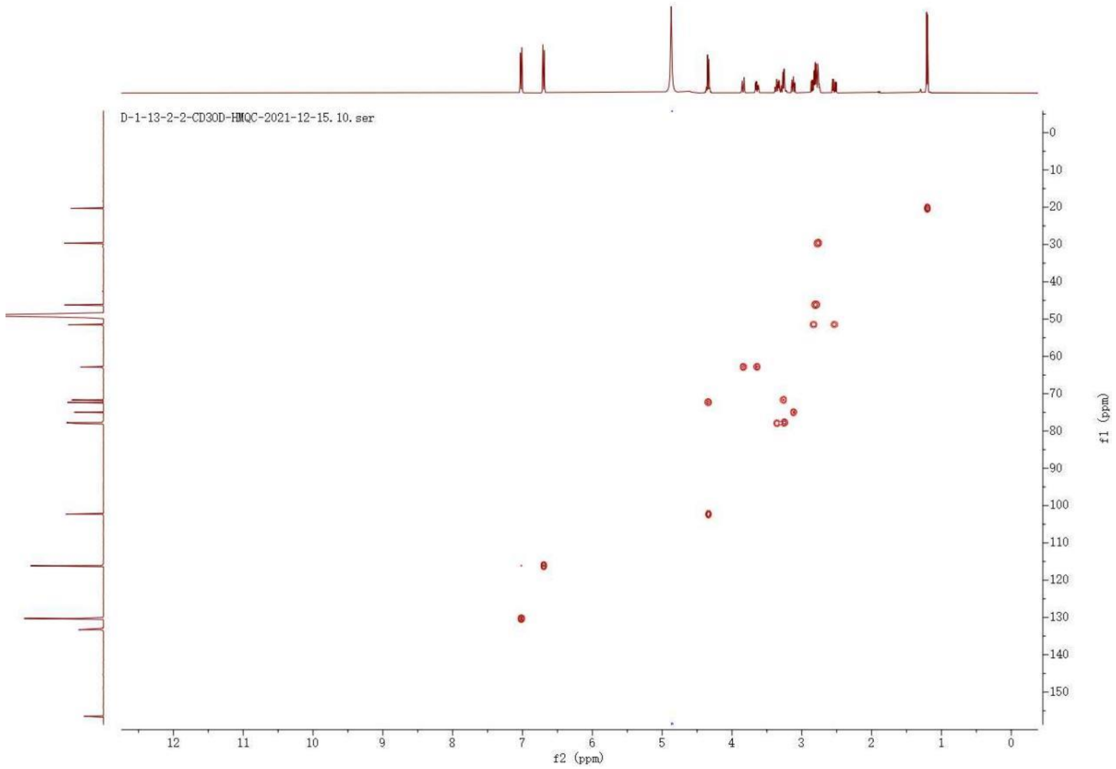

Figure S5-3. HMQC spectrum of **5** in CD_3OD

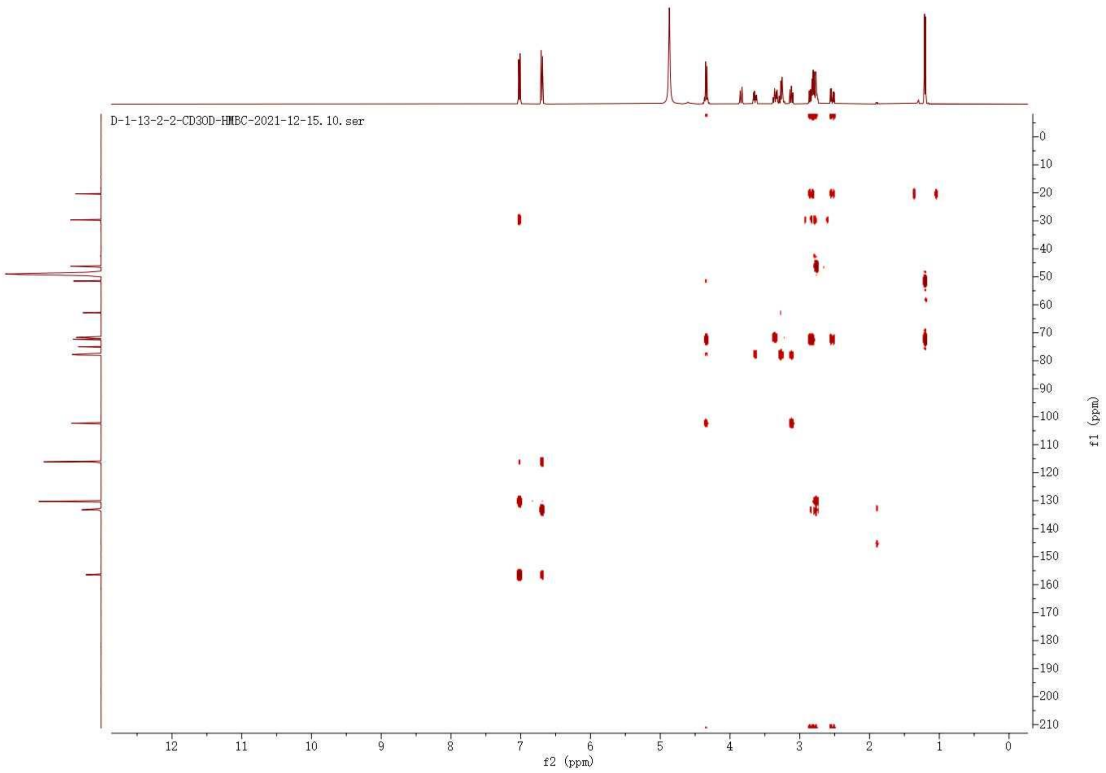

Figure S5-4. HMBC spectrum of **5** in CD_3OD

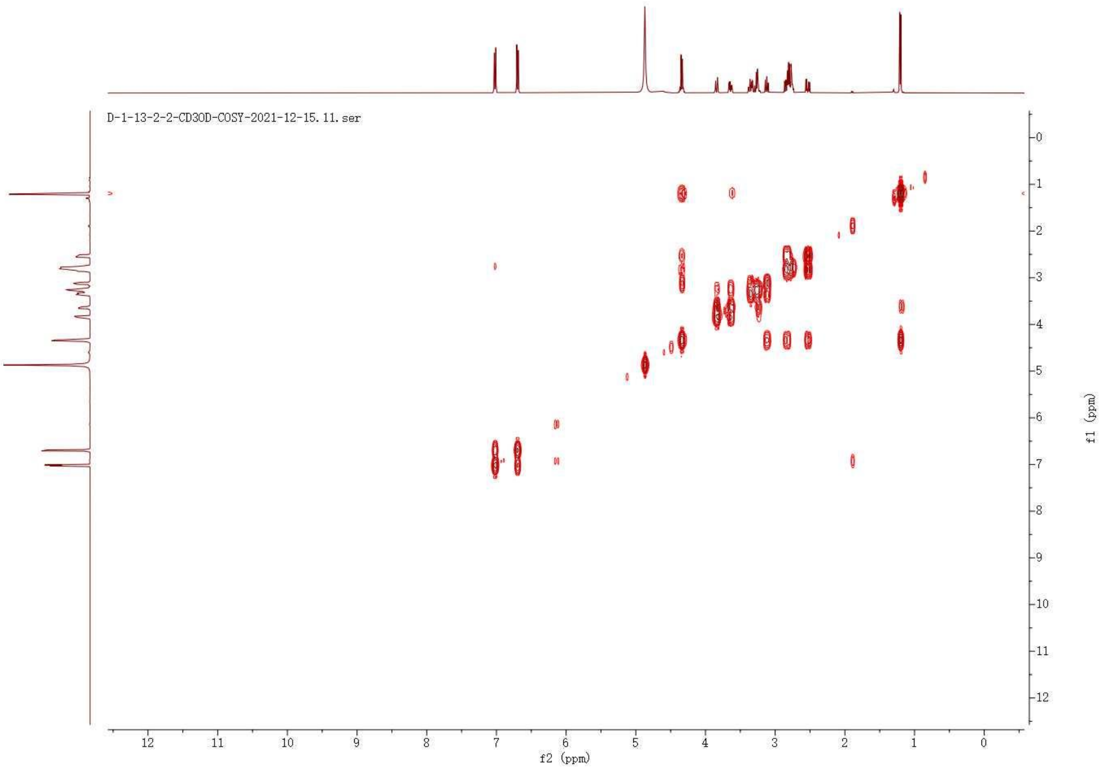

Figure S5-5. ^{1}H-^{1}H COSY spectrum of **5** in CD_3OD

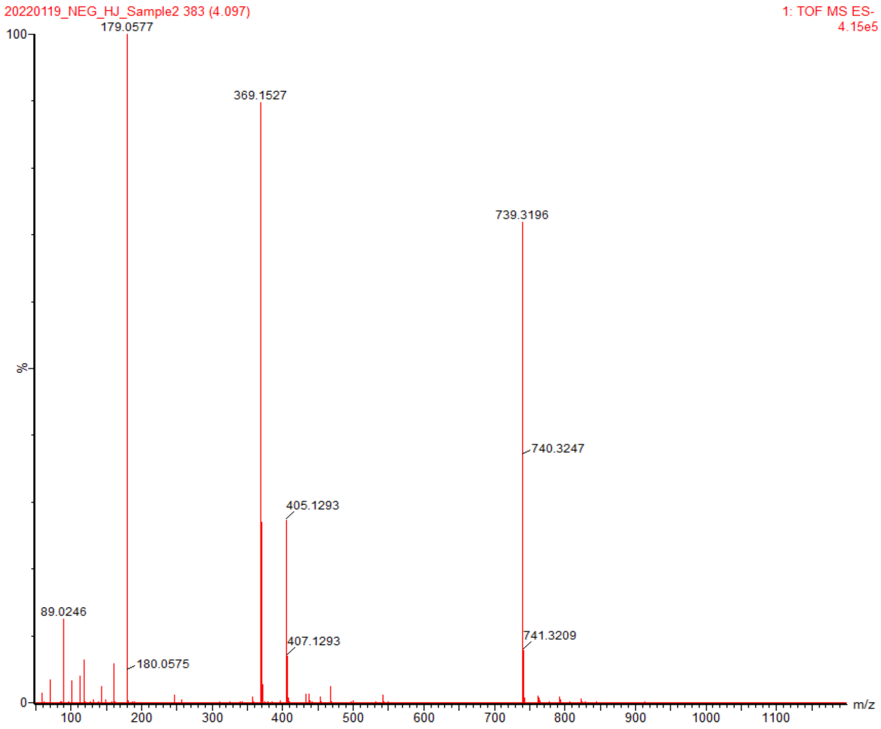

Figure S5-6. HR-ESI-MS spectrum of **5**

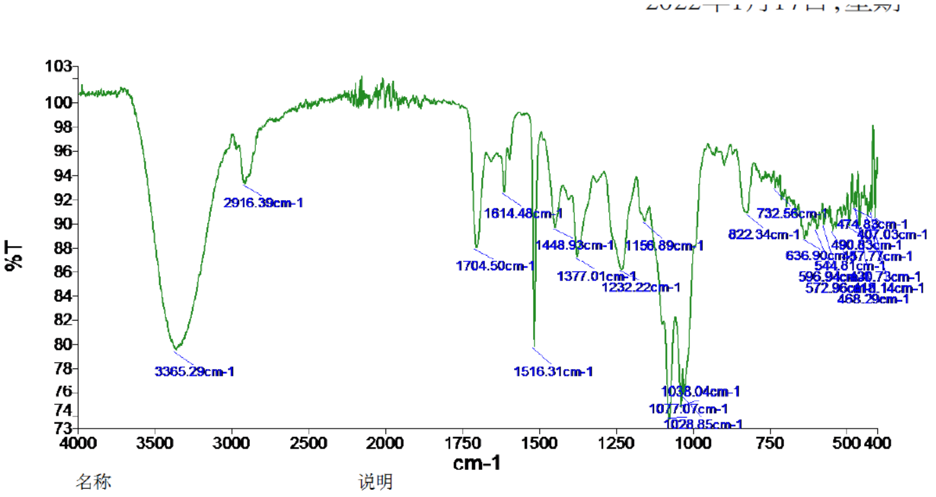

Figure S5-7. IR spectrum of compound **5** (film)

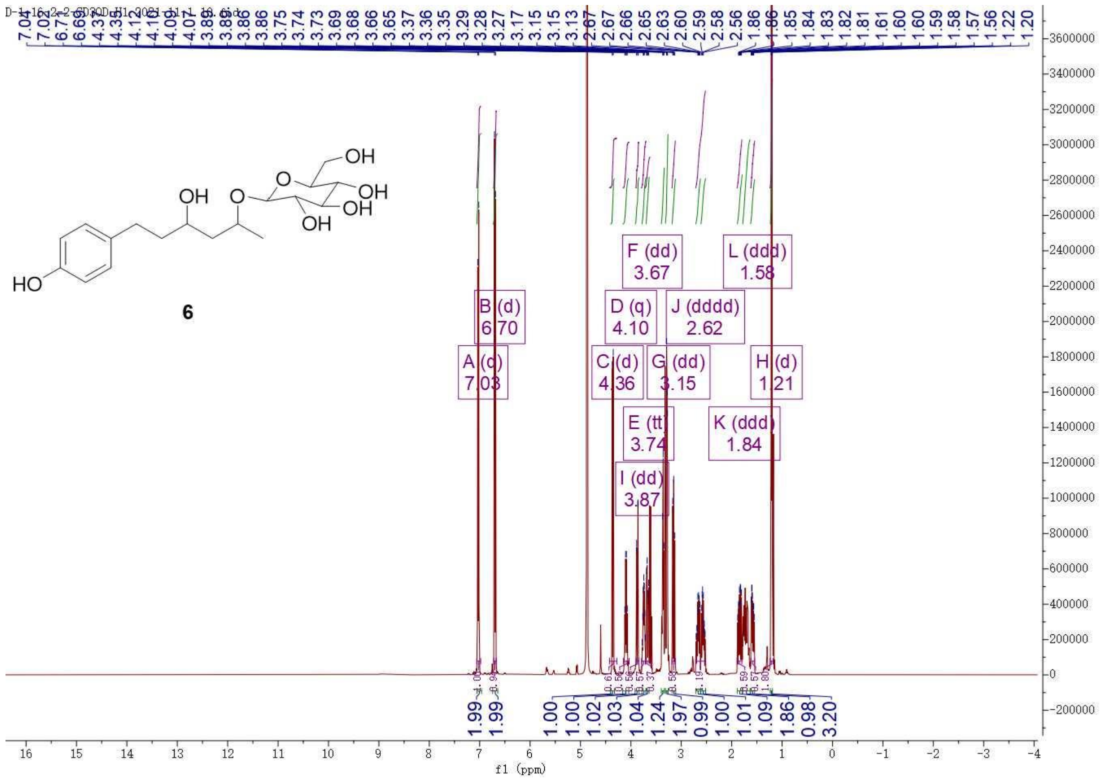

Figure S6-1. ^{1}H-NMR spectrum (400 MHz) of **6** in CD_3OD

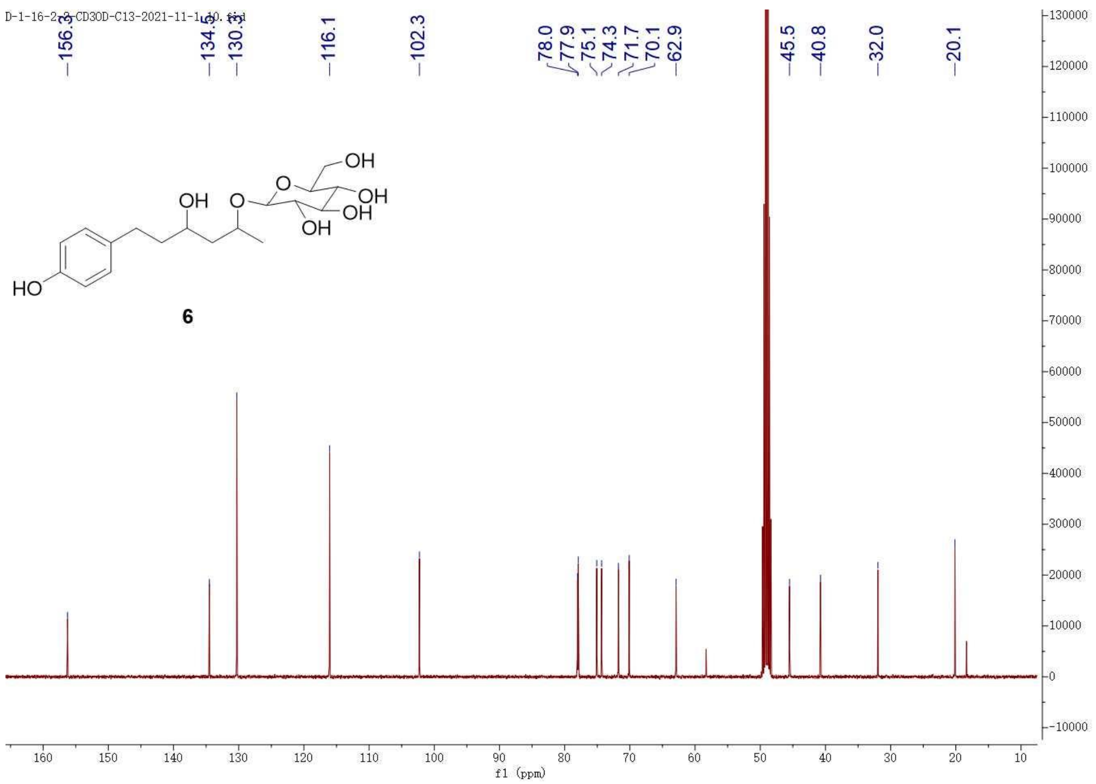

Figure S6-2. ^{13}C-NMR spectrum (100 MHz) of **6** in CD_3OD

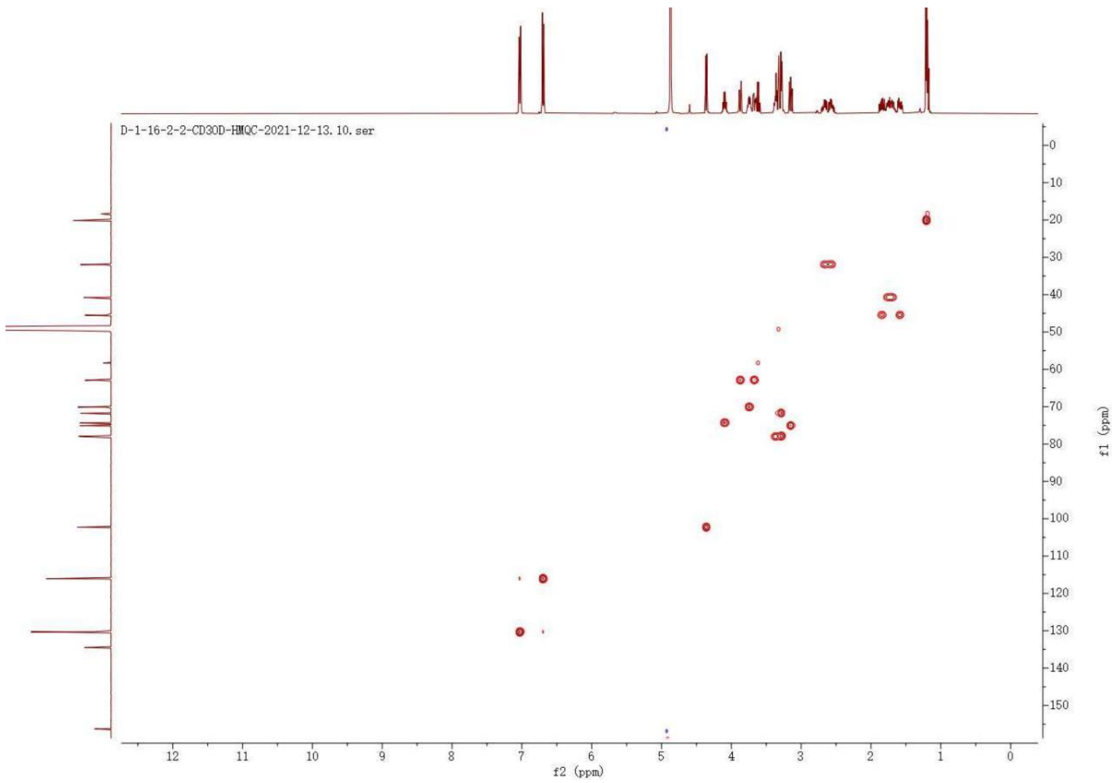

Figure S6-3. HMQC spectrum of **6** in CD_3OD

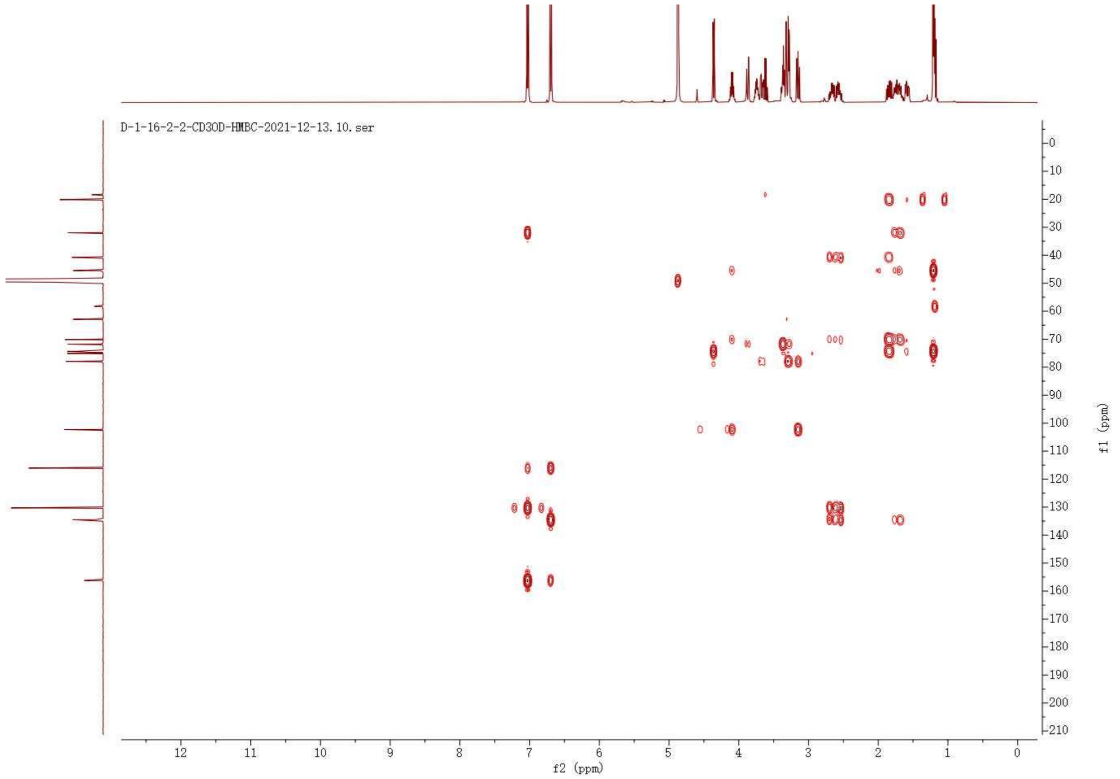

Figure S6-4. HMBC spectrum of **6** in CD_3OD

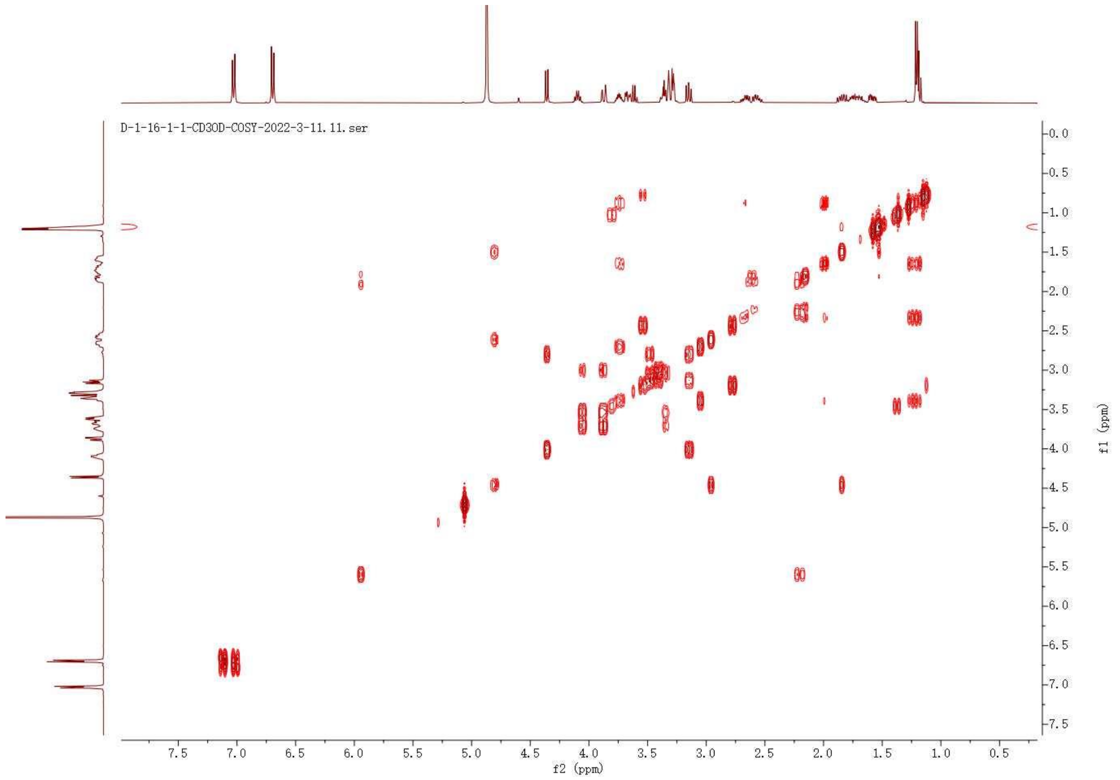

Figure S6-5. ^1H-^1H COSY spectrum of **6** in CD_3OD

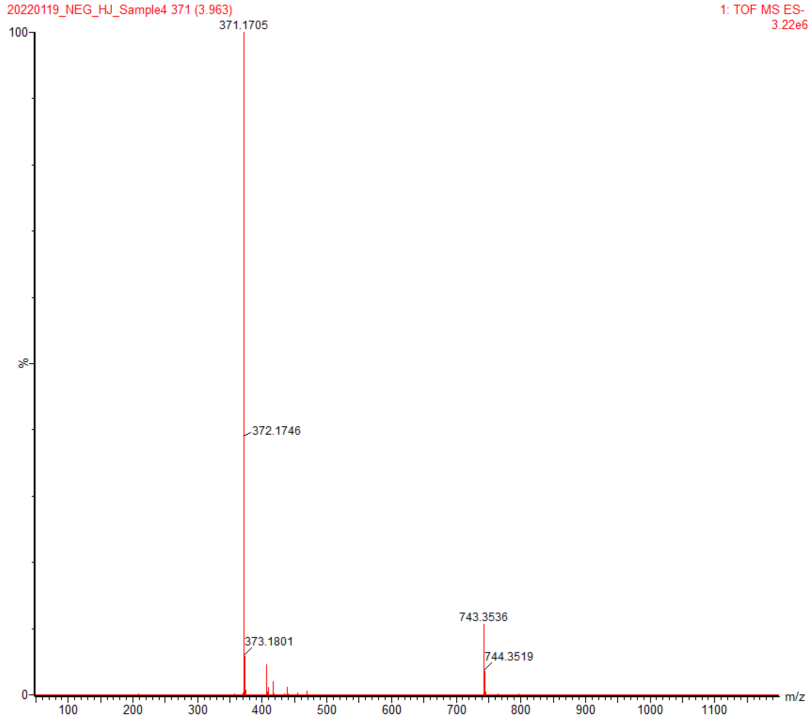

Figure S6-6. HR-ESI-MS spectrum of **6**

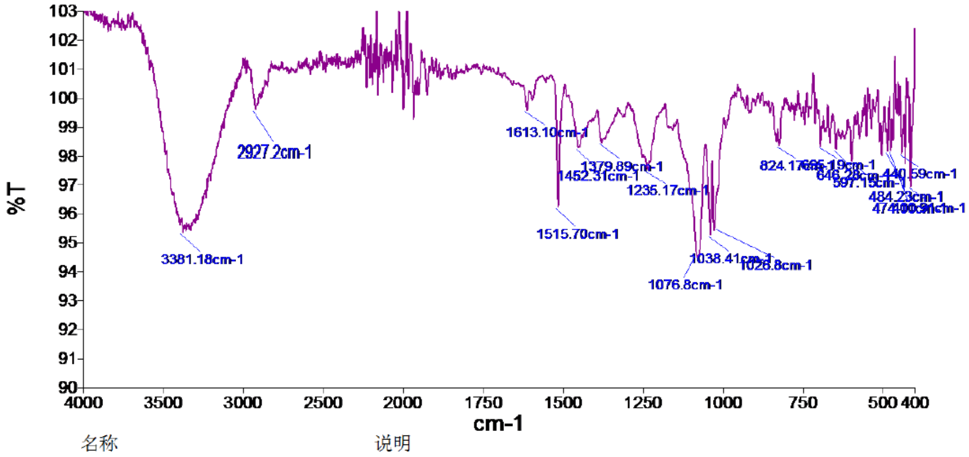

Figure S6-7. IR spectrum of compound **6** (film)

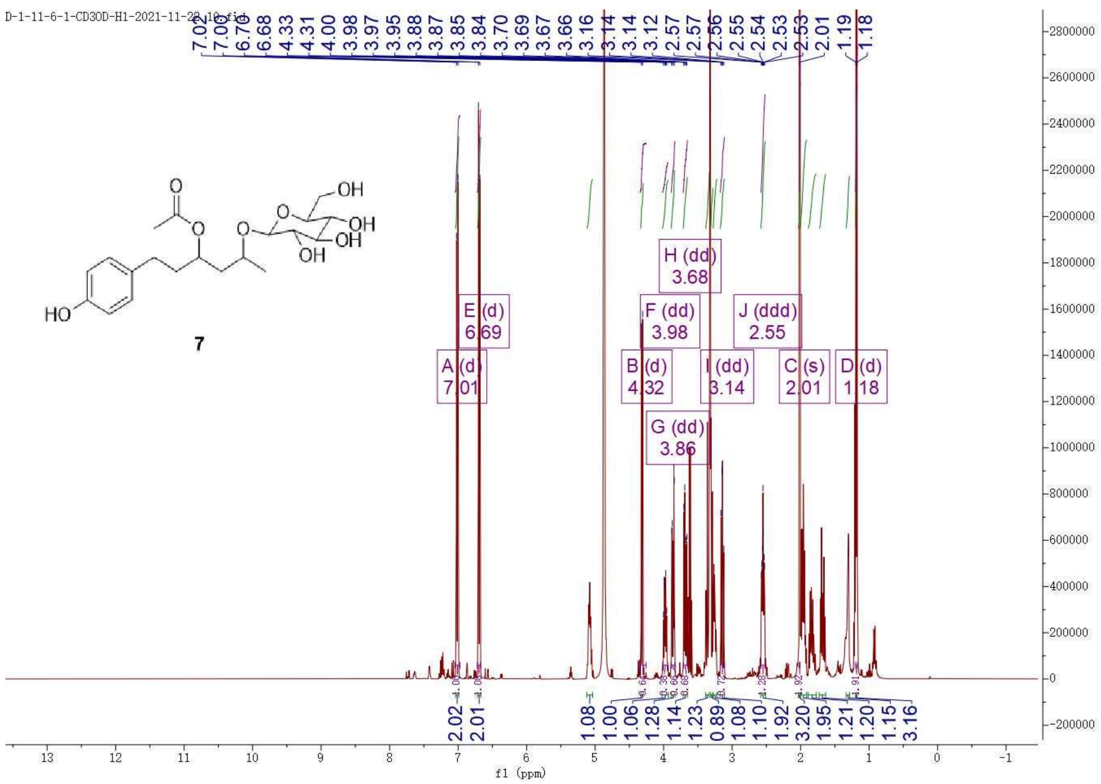

Figure S7-1. ^{1}H-NMR spectrum (400 MHz) of **7** in CD_3OD

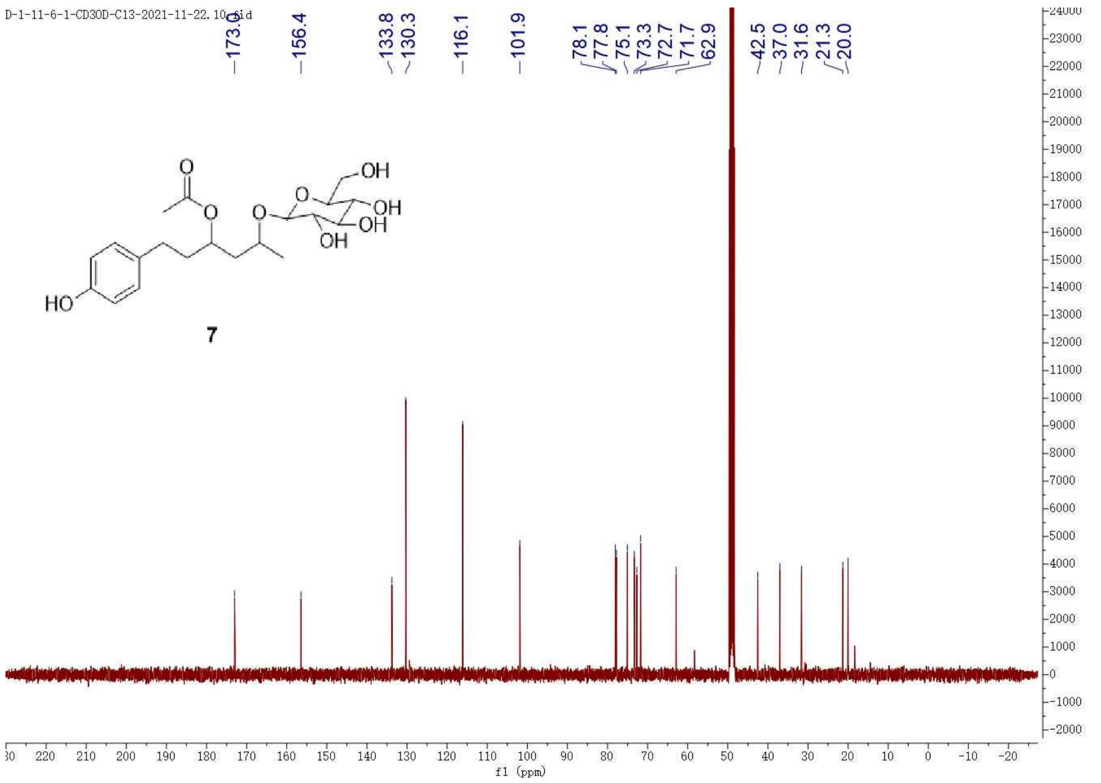

Figure S7-2. ^{13}C-NMR spectrum (100 MHz) of **7** in CD_3OD

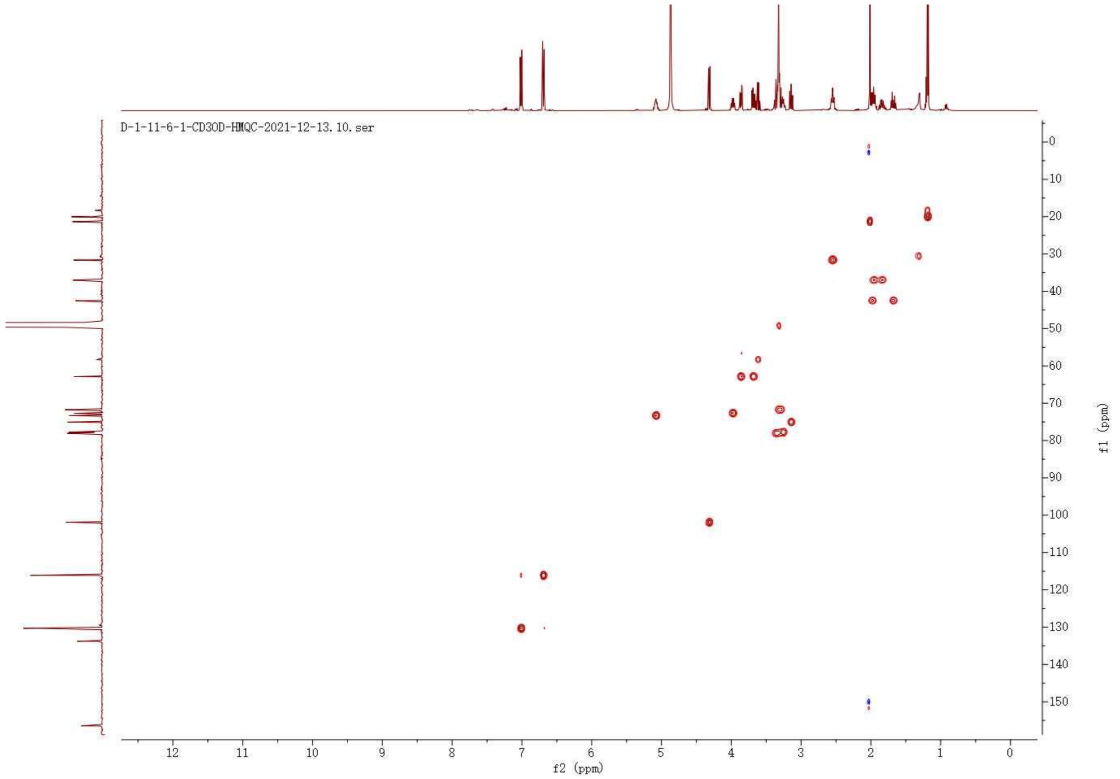

Figure S7-3. HMQC spectrum of **7** in CD_3OD

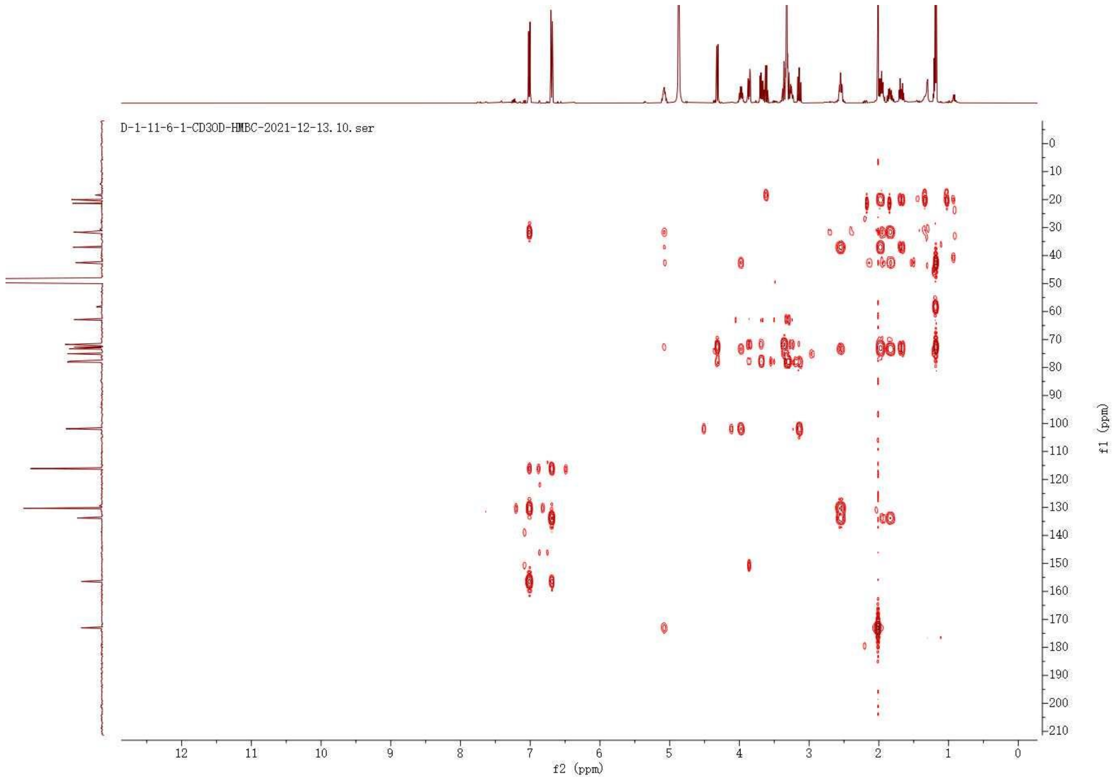

Figure S7-4. HMBC spectrum of **7** in CD_3OD

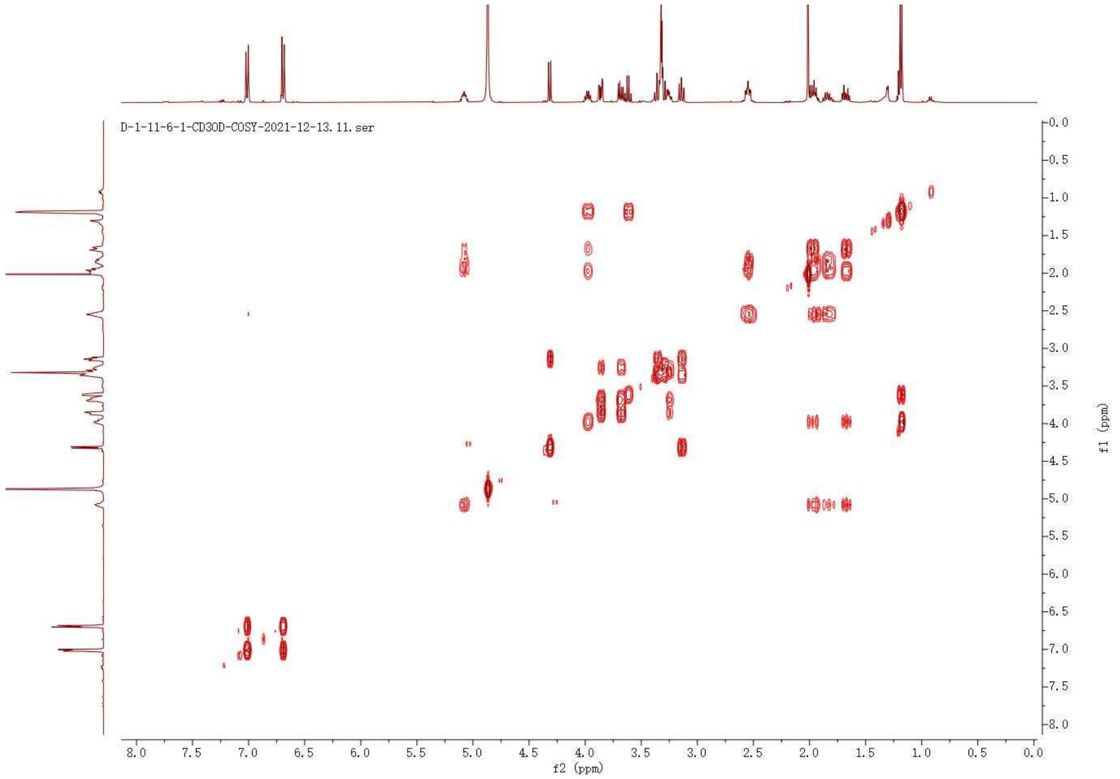

Figure S7-5. ^{1}H-^{1}H COSY spectrum of **7** in CD_3OD

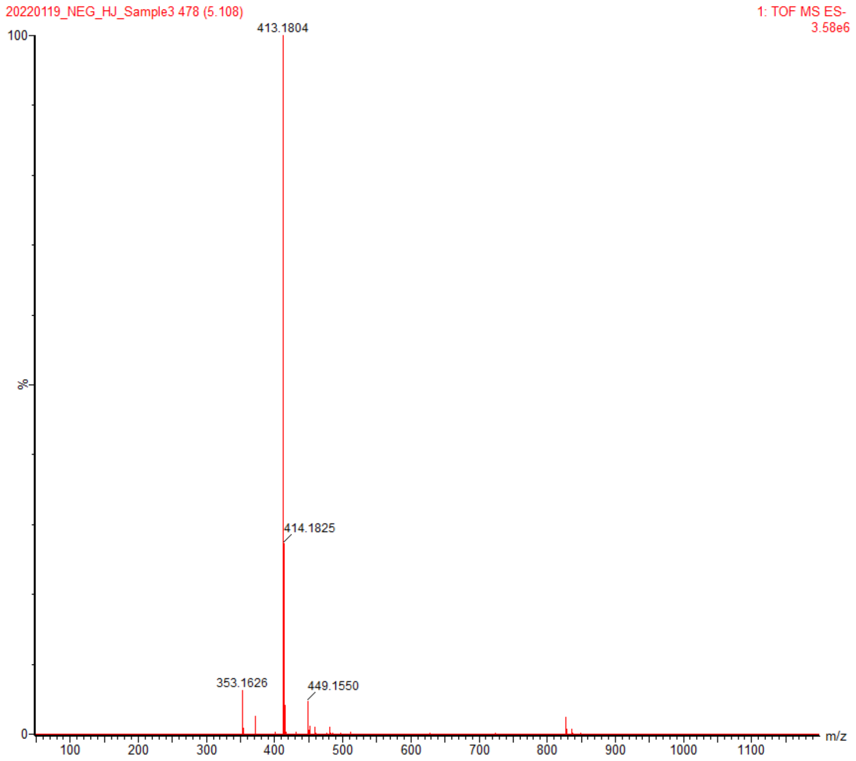

Figure S7-6. HR-ESI-MS spectrum of **7**

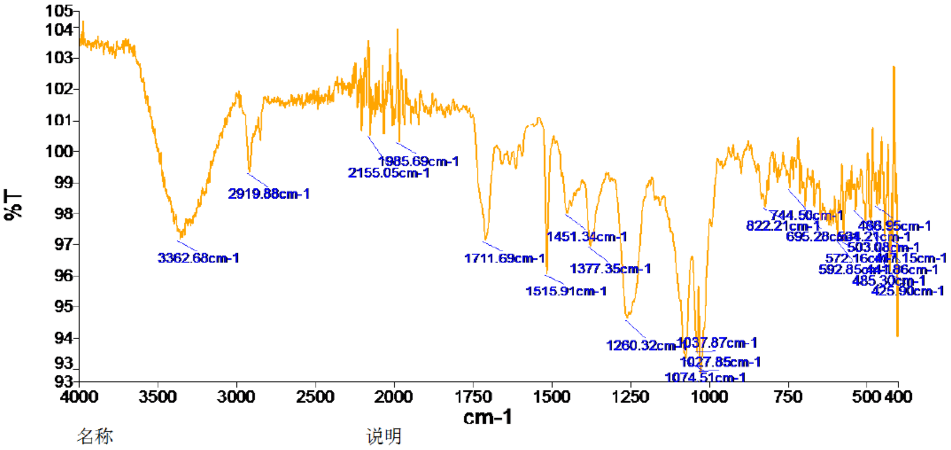

Figure S7-7. IR spectrum of compound **7** (film)
